# Function Coupling Mechanism of PhuS and HemO in Heme Degradation

**DOI:** 10.1038/s41598-017-11907-5

**Published:** 2017-09-12

**Authors:** Michael J. Y. Lee, Ye Wang, Yafei Jiang, Xichen Li, Jianqiu Ma, Hongwei Tan, Keegan Turner-Wood, Mona N. Rahman, Guangju Chen, Zongchao Jia

**Affiliations:** 1 Department of Biomedical and Molecular Sciences, Queen’s University, Kingston, Ontario, K7L 3N6 Canada; 2 College of Chemistry, Beijing Normal University, 100875 Beijing, China

## Abstract

Most bacteria possess only one heme-degrading enzyme for obtaining iron, however few bacteria such as *Pseudomonas aeruginosa* express two, namely PhuS and HemO. While HemO is a well-known heme oxygenase, previously we discovered that PhuS also possesses heme degradation activity and generates verdoheme, an intermediate of heme breakdown. To understand the coexistence of these two enzymes, using the DFT calculation we reveal that PhuS effectively enhances heme degradation through its participation in heme hydroxylation, the rate limiting reaction. Heme is converted to verdoheme in this reaction and the energy barrier for PhuS is substantially lower than for HemO. Thus, HemO is mainly involved in the ring opening reaction which converts verdoheme to biliverdin and free iron. Our kinetics experiments show that, in the presence of both PhuS and HemO, complete degradation of heme to biliverdin is enhanced. We further show that PhuS is more active than HemO using heme as a substrate and generates more CO. Combined experimental and theoretical results directly identify function coupling of this two-enzyme system, resulting in more efficient heme breakdown and utilization.

## Introduction

Heme oxygenase (HO) is a family of enzymes which catalyze the degradation of heme to CO, free iron and biliverdin^1^. These degradation products play important physiological roles in cells. In pathogenic bacteria, HO is also indispensable for protecting the bacteria from the toxicity of heme, and acquiring iron from the host for proliferation due to very limited soluble iron. Therefore, HO is abundant in nearly all classes of eukaryotes and prokaryotes^1–6^. Typical heme oxygenases, such as HO-1 and HO-2 from mammals^7^, HmuO in *Corynebacterium diphtheriae* ^8^, HemO in *Neisseria meningitidis* ^9^, all share similar folds and utilize a similar mechanism to degrade heme. The active site of HO binds heme between the proximal and distal helices in a sandwich manner. On the proximal side is a conserved histidine residue that coordinates the iron of heme, while the distal residues are different in various HOs.

**Figure 1:**
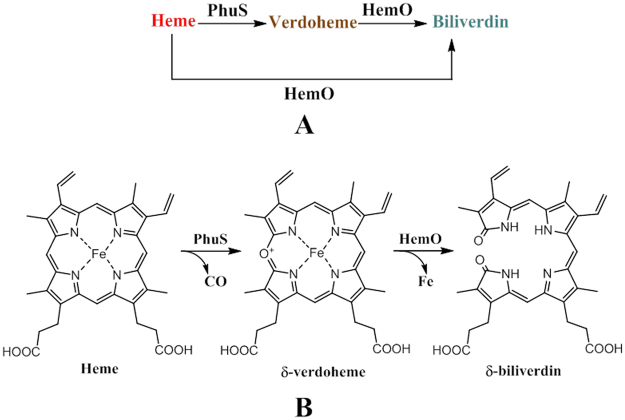
Heme degradation in *P*. *aeruginosa*. (**A**) Heme can take one of two degradation pathways once in the cytoplasm of *P*. *aeruginosa*. The pathway involving both PhuS and HemO is likely to occur most frequently. (**B**) Simplified schematic of heme degradation involving PhuS and HemO. PhuS catalyzes the multiple steps involved in the degradation of heme to verdoheme, releasing CO in the process. HemO catalyzes the conversion of verdoheme to biliverdin, releasing free iron.

Most bacteria contain only one heme degradation enzyme to obtain iron; nevertheless a few bacteria including the opportunistic human pathogen *Pseudomonas aeruginosa* encode two iron-regulated enzymes implicated in heme breakdown, namely PhuS and HemO^10^. *P*. *aeruginosa* also encodes a separate heme oxygenase, BphO, which is not iron-regulated and is used to produce a different isomer of biliverdin than HemO for use as a chromophore for the sensor kinase BphP^11^. While HemO is a well-known heme oxygenase similar to other canonical HOs, PhuS was once considered a trafficking protein that specifically transfers heme to HemO for heme breakdown. However, recently we discovered that PhuS also possesses heme degradation activity. In the presence of an electron donor and catalase, PhuS was observed to degrade heme to verdoheme, an intermediate of heme breakdown^10^. However, HemO *alone* can break down heme in the absence of PhuS *in vitro* ^10^ and *in vivo* ^12^, and PhuS knockout mutants are still able to survive but do not grow as efficiently as wild type *P*. *aeruginosa* ^12^. HemO enables complete degradation of heme to biliverdin. Thus, the intermediates of this process, including verdoheme, are not detectable. In the absence of HemO *in vivo*, specific biliverdin isomers produced by HemO were not observed^13^; however there has been no report showing that verdoheme is not produced by a HemO deletion strain. Because of the fact that HemO alone is able to break down heme and PhuS also possesses heme degradation activity, it has been difficult to comprehend the coexistence of two heme-degrading enzymes within the same heme utilization pathway. We had previously proposed that PhuS may degrade the heme partially to verdoheme, and transfer the verdoheme, instead of heme, to HemO for subsequent degradation to produce biliverdin^10^ (Fig. 1). As such, heme degradation would be accomplished by two enzymes as opposed to one canonical HO. An important question arising from this hypothesis is what, if any, advantage might there be for this two-enzyme system.

## Results and Discussion

**Figure 2:**
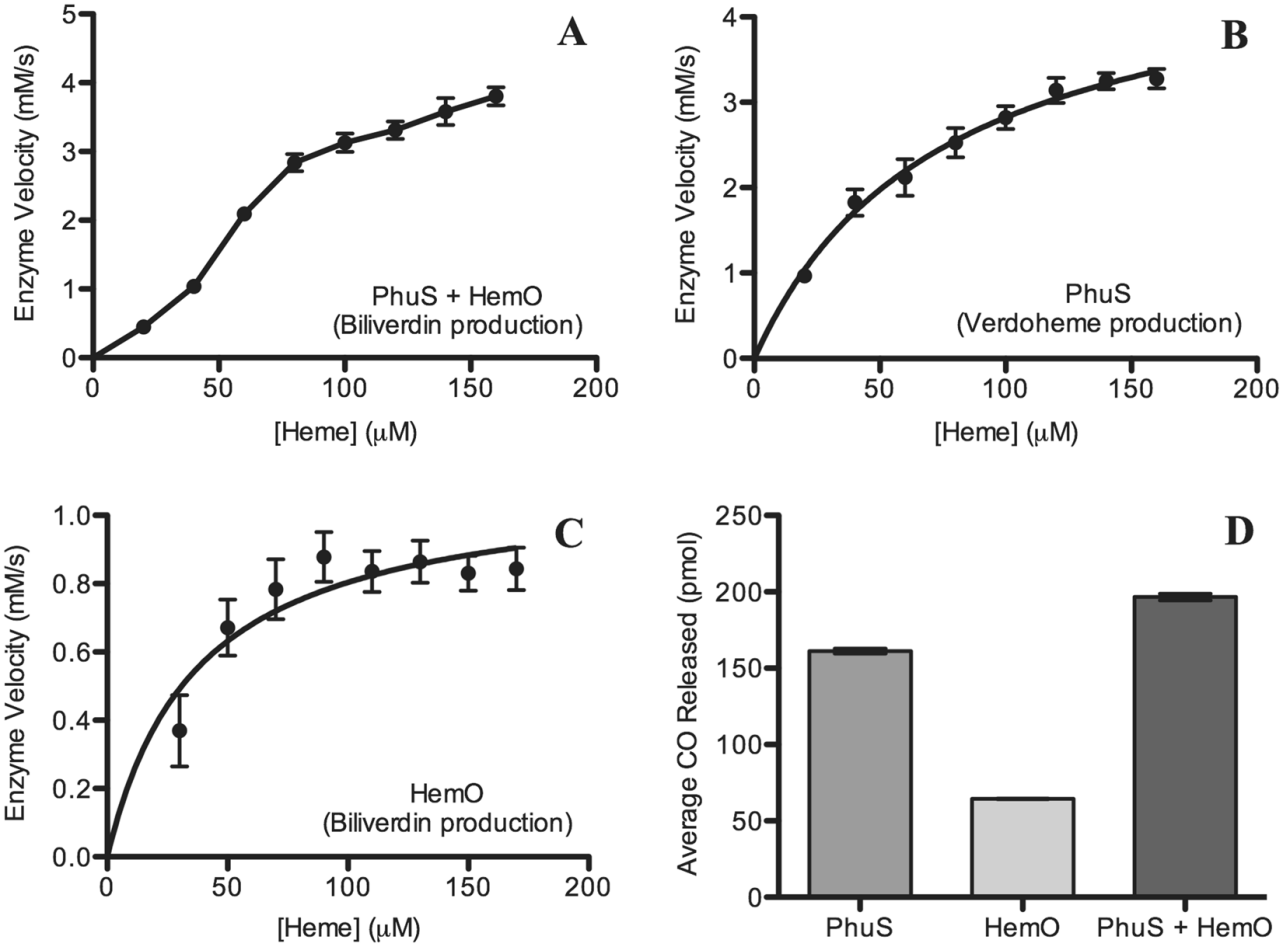
PhuS and HemO together evidently enhances heme degradation. (**A**) Final velocity plot of biliverdin production for PhuS and HemO together obtained by averaging velocity values of five replicates for each reaction. Each reaction contained 40 μM of both enzymes in the presence of increasing heme concentrations. (**B** and **C**) Final velocity curves for PhuS (**B**) and HemO (**C**) obtained by averaging velocity values of five replicates for each reaction. Each reaction contained 40 μM enzyme in the presence of increasing heme concentrations. Error bars represent standard error of the mean. Verdoheme production was monitored at 655 nm for PhuS (**B**), and biliverdin at 671 nm was monitored for PhuS + HemO (**A**) and HemO (**C**). (**D**) Comparison of the average amount of CO released by 0.1 μM of PhuS, HemO, and PhuS + HemO mixture. 50 μM heme was used for each reaction, along with 50 μM ascorbic acid to initiate the reaction.

To answer the aforementioned question, in this work we sought to reveal in the presence of PhuS, whether HemO’s heme degradation activity would be altered using a combined approach of experimental and theoretical investigation. We developed a rapid and simple method to study the kinetics of enzyme-catalyzed heme degradation using apo-enzymes (instead of heme-conjugated). We expressed and purified PhuS and HemO as per a previously published protocol^10^. Fresh heme solution was mixed with protein solution and reactions were initiated by adding L-ascorbic acid (detailed experimental procedures found in Materials and Methods). Cytochrome P450 reductase-NADPH has also been previously shown to initiate heme degradation by PhuS^10^. To compare HemO’s activity in the presence and absence of PhuS, two protein samples were used, namely HemO and PhuS together (both 40 μM) and HemO alone (40 μM). Absorbance at 671 nm for biliverdin was measured to monitor the amount of product formed, following titration of increasing amounts of heme. As shown in Fig. 2, the production of biliverdin in the two-enzyme system (HemO + PhuS) is faster than HemO alone (Fig. 2A,C). At a heme concentration of 160 μM, PhuS and HemO together produced biliverdin more than four times faster. These results show that in the presence of PhuS, overall heme degradation is clearly enhanced. Furthermore, the velocity plot of HemO + PhuS appears to be sigmoidal, in contrast to HemO alone. Thus, it appears the two-enzyme system experienced an initial limiting step and exhibited cooperativity as the reaction proceeded, revealing considerable differences between the two systems (HemO + PhuS *vs*. HemO).

To understand HemO’s lower catalytic efficiency than the HemO + PhuS system, as well as compared to canonical HOs such as HmuO^14^, we investigated the mechanism of HemO and PhuS catalyzed heme degradation with ONIOM(UB3LYP:AMBER) calculations. Chemical models were constructed from the 1.6 Å-resolution crystal structure of heme-bound HemO (PDB code: 1SK7)^15^ and 1.95 Å-resolution crystal structure of heme-bound PhuS (PDB code: 4MF9)^10^. Although earlier reports showed that the HO enzyme exclusively utilizes O_2_ for verdoheme degradation, further experiments indicated that HOs could utilize either H_2_O_2_ or O_2_ to complete heme degradation, and the surrogate pathway with H_2_O_2_ as the oxidant is faster than the native pathway with O_2_^16^. Since previous studies revealed that H_2_O_2_ and O_2_ follow a similar mechanism^14^, in this work only H_2_O_2_ as the oxygen source was investigated. The ONIOM method was employed to explore the reaction mechanism^17^. UB3LYP functional was used in conjunction with the LANL2DZ effective core potential basis set for iron and the 6–31 G* basis set for other atoms in the QM region^18, 19^. The remaining atoms were treated as the MM region with AMBER ff98^20^.

**Figure 3:**
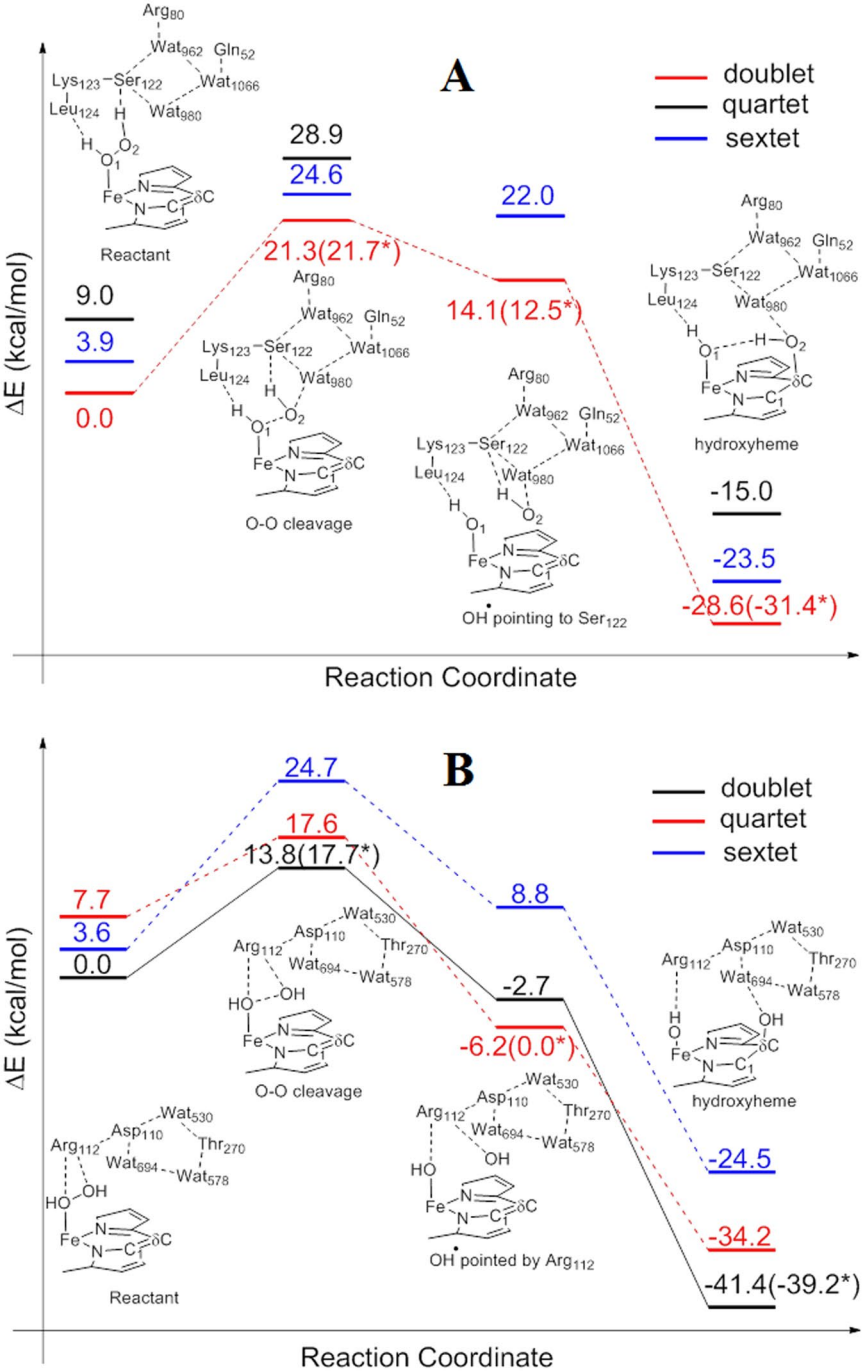
Energy diagrams with schematic descriptions of the characteristics of the reaction species. (**A**) HemO-catalyzed heme hydroxylation, which is rate-limited by O-O bond cleavage with a 21.3 kcal·mol^−1^ energy barrier. (**B**) PhuS-catalyzed heme hydroxylation, which follows the same reaction mechanism but only experiences a 13.8 kcal·mol^−1^ high energy barrier. *The energy barrier (shown in brackets) for the rate-limiting step is further refined using B3LYP functional with LANL2DZ for iron and cc-pVTZ for the remaining atoms.

**Figure 4:**
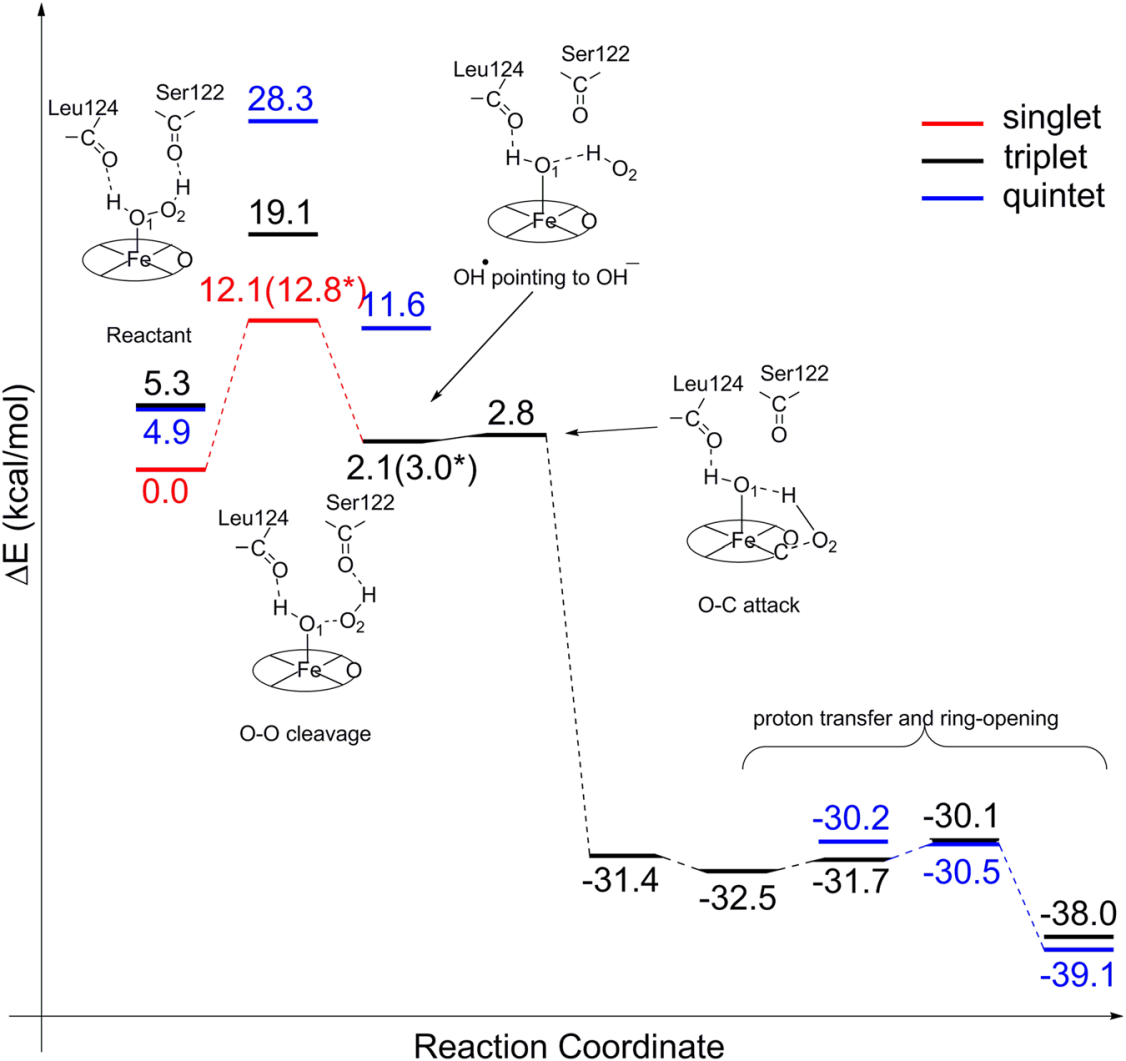
Energy diagram of HemO-catalyzed verdoheme ring cleavage. The reaction is also rate-limited by O-O bond cleavage with 12.1 kcal·mol^−1^ energy barrier. *The energy barrier (shown in brackets) for the rate-limiting step is further refined using B3LYP functional with LANL2DZ for iron and cc-pVTZ for the remaining atoms.

Heme degradation is completed through two catalytic reactions. In the first reaction, which can be divided into two steps, heme is firstly hydroxylated on a *meso* position carbon to form the *meso*-hydroxyheme; the hydroxyheme is then converted to verdoheme and CO. Finally, the verdoheme macrocycle is cleaved into biliverdin, releasing free ferrous iron. It is believed that the heme hydroxylation step and verdoheme cleavage step are completed enzymatically, while the conversion of hydroxylated heme to verdoheme proceeds spontaneously^21^. The reaction energy profiles calculated for the heme hydroxylation and verdoheme ring cleavage reactions by HemO are presented in Figs 3 and 4. The O-O bond cleavage is rate-limiting in both reactions, which is not surprising considering that two sequential reactions take place in a single active center of HemO. Yet the energy barriers are very different in the two reactions, 21.3 kcal·mol^−1^ for heme hydroxylation and 12.1 kcal·mol^−1^ for verdoheme cleavage respectively. Given the rather significant difference in energy barrier, it appears that HemO is specialized in cleaving the O-O bond during verdoheme cleavage but not heme hydroxylation.

Heme hydroxylation by HemO is initiated by O-O bond cleavage. Along with O-O bond elongation, the proximal OH group (ligating Fe) obtains an electron from the Fe(III)-porphyrin complex and forms hydroxide, while the distal OH radical becomes stabilized by the hydrogen bond network inside the active pocket, which is formed among three structural water molecules (Wat962, Wat980, Wat1066) and three residues Ser122, Arg80, Gln52 (as seen from the corresponding geometric structures in Supporting Information). A hydrogen bond network involving structural water molecules is ubiquitous for HOs^7, 22–32^, which has been identified to play indispensable roles in the heme degradation reaction^14^. After critical O-O bond homolysis of H_2_O_2_, the resulting OH radical attacks the *meso*-C atom of heme without any barrier. In contrast, a prerequisite transition state involving flipping of the OH radical was reported for HmuO^14^.

O-O bond cleavage during the verdoheme ring opening reaction shares most of the structural characteristics with those of heme hydroxylation. The hydrogen bond network stabilizes OH radicals in the transition state. Small differences in Fe-O coordination and O-O distance were observed between the two transition states (corresponding geometric coordinates and figures in Supporting Information), reflecting changes of oxidation states of iron proceeding from heme to verdoheme. After O-O bond homolysis, the subsequent reaction process is controlled by the hydrogen bond network, which guides the movement of the distal OH radical and ensures its optimal orientation for an eventual attack on the pyrrole position of the verdoheme ring.

Judging from our DFT calculations, HemO shares most characteristics of its heme degradation mechanism with canonical HOs, such as O-O cleavage being the rate-limiting step, and the hydrogen bond network playing a critical role in the reaction such as stabilizing the transition states. The low efficiency of HemO compared to HmuO^12^ lies in its relative higher energy barrier of homolytic cleavage of the O-O bond in heme hydroxylation. It is worth noting that HemO has a very compact distal pocket, where the distal α-helix is located very close to the heme plane so that H_2_O_2_ serves as hydrogen bond donors to form two hydrogen bonds with the backbone carbonyls of Ser122 and Leu124. Though these two hydrogen bonds assist the binding of H_2_O_2_ in the active center, they also suppress the activity of H_2_O_2_ as an oxidant, since H_2_O_2_ pulls electronic density toward itself through these two hydrogen bonds. While in HmuO, H_2_O_2_ forms only one accepting hydrogen bond with a structural water molecule, which, in contrast, promotes the activity of H_2_O_2_.

Our DFT calculation results also indicate that HemO is highly efficient in the verdoheme cleavage reaction step. This observation is in accordance with our proposed mechanism in which HemO is the preferred enzyme that converts verdoheme to biliverdin in the HemO-PhuS two-enzyme system. The determining factor for the significant enhancement in HemO-catalyzed verdoheme ring opening compared to heme hydroxylation stems from the intrinsic difference of redox partners involved. The hydrogen bonding network only experiences slight changes in these two reactions. As a result, the oxidant H_2_O_2_ can be considered comparably capable of electron salvage in both processes, which is evident from the Mulliken spin density populations (Table 1). In the hydroxylation and verdoheme cleavage reactions, the spin density change of the oxidant H_2_O_2_ between the reactant and transition states are 0.49 and 0.45 respectively. On the other hand, the reductants in the two reactions are different. In the verdoheme ring opening reaction, ferrous Fe^2+^ acts as the electron provider and its spin population changes from 0.00 in the reactant state (RE) to −0.46 in the transition state (TS). While in the hydroxylation step, porphyrin ring plays the role as the reductant in the O-O bond homolysis instead. Porphyrin is obviously not as good as ferrous iron in the reducing reaction. The reported porphyrin ring redox potential is approximately 1 V^33^, significantly higher than that of Fe^2+^/Fe^3+^ (0.77 V). The notable difference of the reductant in the two reactions results in very different energy barriers.

**Table 1:** Mulliken spin populations for various species during PhuS and HemO catalyzed heme degradation^#^.

		Fe	${{\bf{O}}}_{{{\bf{H}}}_{{\bf{2}}}{{\bf{O}}}_{{\bf{2}}}}$ (bound)	${{\bf{O}}}_{{{\bf{H}}}_{{\bf{2}}}{{\bf{O}}}_{{\bf{2}}}}$ (unbound)	His*	Por*
PhuS heme hydroxylation	Re	1.02	0.00	0.00	−0.01	0.00
	TS	0.97	−0.05	−0.43	0.00	0.44
	INT	0.91	0.15	0.77	0.00	0.00
HemO heme hydroxylation	Re	1.05	−0.01	0.00	−0.03	−0.01
	TS	0.75	−0.02	−0.47	0.00	0.74
	INT	0.93	−0.06	−0.83	0.00	0.00
HemO verdoheme ring-opening	Re	0.00	0.00	0.00	0.00	0.00
	TS	−0.46	−0.02	0.43	0.00	0.04
	INT	0.93	0.11	0.97	0.00	0.00

^#^The spin density population represents the total electron density of alpha spin electrons minus that of beta electrons of the corresponding atoms. *His is the histidine residue, which coordinates the iron center of heme. Por stands for porphyrin ring.

The ferric to ferrous switch from heme to verdoheme also results in a delicate change in the metal ligation between RE and TS. In heme hydroxylation, given that ferric iron is partially reduced from RE to TS, ligation strength due to H_2_O_2_ homolysis is compromised. While in the verdoheme cleaving step, ferrous iron is oxidized to the ferric state in the TS, which further strengthens the ligation between proximal OH and Fe, thus facilitating the reaction. In fact, in the heme hydroxylation and verdoheme cleaving steps, the Fe-O distance is shortened by 0.18 and 0.24 Å respectively (corresponding geometric coordinates and figures in Supporting Information), demonstrating the effect of the valence change of iron.

To further understand the reason why addition of PhuS accelerates heme degradation, we next examined heme hydroxylation catalyzed by PhuS. Although the reaction follows a very similar stepwise mechanism as that of HemO with the rate-limiting step of O-O homolysis, the energy barrier of 13.8 kcal·mol^−1^ for PhuS is considerably lower than 21.3 kcal·mol^−1^ for HemO, even lower than the barrier of 14.3 kcal·mol^−1^ reported for HmuO^14^. These results suggest that PhuS is a preferred enzyme in catalyzing the first reaction and is specialized in heme oxidation.

**Figure 5:**
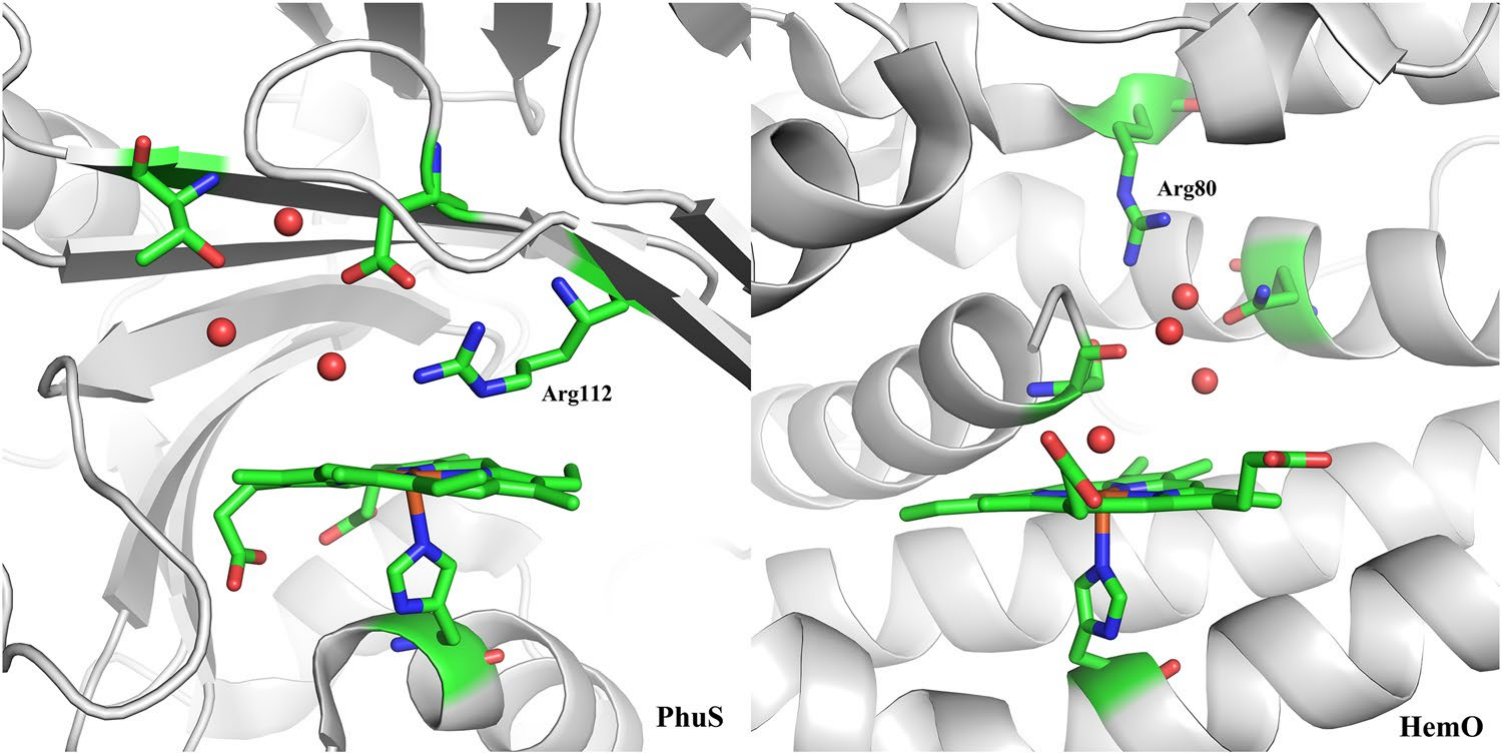
Comparison of the Arg positions in PhuS and HemO. Side chains of the residues near the active site are drawn as sticks. Oxygen atoms of adjacent waters are drawn as balls. In PhuS, Arg resides right above the heme ring (left), while in HemO, there are several residues and water molecules located between Arg and the heme ring (right).

The high activity of PhuS owes to its unique active center structure. One prominent feature of PhuS is that an arginine residue (Arg112) is positioned very close to the heme ring and bisects the heme plane from its distal side (Fig. 5). The positively-charged guanidyl group of Arg112 forms two hydrogen bonds with the H_2_O_2_ which binds Fe. These hydrogen bonds withdraw electrons from H_2_O_2_ and consequently raise its oxidative capability. In contrast, in the active center of HemO, H_2_O_2_ donates two hydrogen bonds to the backbone carbonyls of Ser122, which increases its electron population and results in lower oxidative capability. As a comparison, in HmuO, H_2_O_2_ acts as the acceptor to form a hydrogen bond with one of the structural water molecules, which also increases the redox potential of H_2_O_2_, though to a limited extent. The spin density population analysis confirmed the role of the hydrogen bonds. In PhuS, from reactant to transition state in the O-O bond homolysis step, the spin density change shows spin population transfer from porphyrin to H_2_O_2_, forming a [Fe(III)-porphyrin•^+^] cation in the transition state. However, in HemO, the electron given out by the porphyrin must be reallocated between H_2_O_2_ and Fe(III), as shown in Table 1. The transition state is thus even more energetically uphill and significantly more difficult to reach.

To experimentally validate the computational findings which suggest that PhuS is a more potent enzyme than HemO in catalyzing heme hydroxylation and converting heme to verdoheme, we conducted further kinetics studies with PhuS. The same experimental procedures for HemO were used except that the absorbance at 655 nm characteristic of verdoheme was used, since PhuS generates verdoheme, an intermediate of heme degradation^10^. Analysis by Michaelis-Menten kinetics revealed that the maximum rate of reaction (V_max_) for PhuS at saturating heme concentrations was 4.9 ± 0.4 mM/s which is >4 times than that for HemO (1.1 ± 0.1 mM/s) (Fig. 2B). Evidently, PhuS is a more potent enzyme than HemO using heme as the substrate given that its ability to bind heme is twice as weak as HemO (see below for Michaelis constants and discussion). This is not surprising as our previous experiments also revealed that PhuS exhibits 5-fold higher activity than the HO from *Escherichia coli*, ChuS^10^. Taken together, our results undoubtedly further confirm that PhuS is not only a heme trafficking protein but also a heme degradation enzyme with high efficiency.

Since HemO converts heme to biliverdin and there is no verdoheme accumulation^10^, we sought to monitor verdoheme production at 655 nm in the presence of both PhuS and HemO. As shown in Supplementary Figure S1, a smaller amount of verdoheme was observed than PhuS alone (Fig. 2B), which is expected since in the PhuS + HemO mixture, HemO will sequester the verdoheme for subsequent verdoheme cleavage, decreasing the amount of verdoheme present in the reaction mixture. This result provides further support for the function coupling in the two-enzyme heme degradation system. Although the difference in energy barrier for heme hydroxylation between PhuS and HemO does not directly correspond with our experimental finding that PhuS degrades heme > 4 times faster than HemO, we simply wanted to see that there indeed was a difference in enzyme velocity, displaying the same trend as our theoretical findings. A possible explanation for the discrepancy is that our *in vitro* experiments may encounter some extraneous factors that would not be accounted for in our theoretical calculations, such as proper buffer conditions, potential product inhibition, *etc*.

To directly compare the catalytic efficiency of the two enzymes, we next focused on the first reaction which can be examined in isolation; this reaction produces CO and verdoheme. However, comparing production of verdoheme is not practical as it is quickly converted to biliverdin in the case of HemO. We thus measured the amount of CO using gas chromatography. As shown in Fig. 2D, PhuS generated ~2.5 times more CO than HemO, once again supporting the notion that PhuS is more active than HemO in the heme hydroxylation reaction. Interestingly, PhuS and HemO together produced only slightly more CO (~18%) than PhuS alone. Since CO is released as a consequence of the initial degradation step to form verdoheme, this would mean that PhuS accounts for the majority of the CO, and thus verdoheme, produced in the PhuS + HemO mixture. The presence of HemO may allow PhuS to quickly dispose of its bound verdoheme to HemO, allowing more PhuS to be available to bind and initiate degradation of extra heme molecules. Hence, we observe an increase in CO production, but not an additive effect, when HemO is present. HemO now only has to perform a one-step reaction to degrade the verdoheme into biliverdin, as opposed to having to go through the entire degradation pathway from heme to biliverdin, which may account for the increase in its production rate. Thus, our CO results clearly demonstrate that PhuS is a preferred enzyme to react with and partially degrade heme to verdoheme, following which HemO completes degradation to generate final products biliverdin and free iron. These results are in full agreement with our proposed model in which PhuS is a preferred enzyme for heme hydroxylation, the limiting step, and HemO completes the final degradation step.

Finally, we varied the ratio of PhuS and HemO in our kinetics experiment. When the amount of PhuS was more than HemO, biliverdin production was increased (Supplementary Figure S2A). In comparison, when the amount of HemO was more than PhuS, biliverdin production did not increase but in fact decreased (Supplementary Figure S2B). This is not surprising because more PhuS generates more verdoheme, the product of the limiting first step, which is quickly converted to biliverdin by HemO. Based on our kinetics results (Fig. 2B,C), the heme concentration at which the reaction rate is half of V_max_ (Michaelis constant, K_m_) for PhuS was 75 ± 13 μM, and for HemO was 37 ± 13 μM, revealing that HemO exhibits a higher affinity for heme. As a less efficient enzyme in the first step of heme degradation, HemO appears to serve as a competitive inhibitor for PhuS. With higher binding affinity, HemO competes with PhuS for heme, reducing the rate of verdoheme production by PhuS and slowing the overall reaction down.

In summary, by combining theoretical and experimental approaches we have shown that PhuS and HemO constitute an efficient two-enzyme system for heme degradation. In the first reaction, PhuS is effective in hydroxylating heme and partially degrading heme to generate CO and verdoheme, the latter of which is readily converted to biliverdin and free iron by HemO. This function coupling results in more efficient heme breakdown. The enhanced heme degradation efficiency afforded by the two-enzyme system offers opportunistic bacteria such as *P*. *aeruginosa* an advantage in protecting itself against heme toxicity and in acquisition of iron, which is very scarce in soluble form, but absolutely required for bacterial cell proliferation and pathogenesis.

## Materials and Methods

### Theoretical Methods and Models

We investigated the mechanism of HemO and PhuS catalyzed heme degradation with ONIOM (UB3LYP:AMBER) calculation using the mechanical embedding approach. Chemical models were constructed from the 1.6 Å X-ray crystal structure of HemO (PDB code: 1SK7) and 1.95 Å X-ray crystal structure of PhuS (PDB code: 4MF9), respectively. H_2_O_2_ was positioned by replacing the water molecule coordinating iron in the crystal structure. UB3LYP functional^19^ was used in conjunction with the LANL2DZ^34–36^ effective core potential basis set for Fe and the 6–31 G* basis set for the rest atoms in the QM region. Frequency calculations were performed at the same level as geometry optimization to verify local minima and transition states and obtain zero-point corrections. All the calculations were performed using the Gaussian 09 package^37^.

For HemO, the QM region of our model consists of the heme ring, the proximal ligand histidine (His15), the residues and water molecules that are involved in the hydrogen bond network in the distal pocket, which include Arg80, Gln52, Ser122, Wat1063, Wat1066, Wat980 and Wat962. Lys132 is also included in the QM part, which forms a salt bridge with one of the propionates on the γ side of heme. The other γ-propionate of heme was manually protonated. The protonation state of all amino acid residues was assigned based on pH 7.0. The QM-MM layer boundary was captured with hydrogen atoms. The whole QM region includes about 162 atoms. The rest part of the HemO was treated as the MM region with AMBER ff98. During optimization, all the atoms in the MM region except those on the QM/MM boundaries were frozen.

For PhuS, since the X-ray structure is in the form of a dimer with two structurally repeating chains of PhuS, only the protein chain A complexed with heme was taken for investigation. The QM region in this study is comprised of the heme with its proximal ligand His209, three residues Asp110, Arg112, Thr270 and three structural water molecules (Wat530, 578, 694) which form the hydrogen bond network in the distal pocket. Two other arginines (Arg222, Arg334) were also included in the QM part, which interact with the carboxylate groups on the γ side of heme via salt bridges. The protonation state of all amino acid residues was assigned based on pH 7.0. The whole QM region includes about 150 atoms. The rest part of the protein was treated with the MM method. Hydrogen atoms were used to cap the cutting boundary between the QM and MM parts. During optimization, all the atoms in the MM region except those on the QM/MM boundaries were frozen.

### Experimental Procedures

In this work, we developed a rapid and simple approach using apo-enzymes to study the kinetics of heme degradation while monitoring product formation, as opposed to using heme-conjugated proteins. In order to understand the presence of two heme-degrading enzymes within the same heme utilization pathway, we applied this approach to the analysis of heme breakdown with PhuS and HemO, both individually and in combination, which, for the first time, revealed PhuS-HemO function coupling.

### Protein Purification and Heme Degradation Experiments

We expressed and purified PhuS and HemO according to a previously published protocol^10^, and carried out heme degradation experiments. Reactions were set up in individual wells of a 96-well plate (Corning Incorporated) with a total volume of 100 μL containing 40 μM purified apo-enzyme in 25 mM Tris pH 8.5, 100 mM NaCl, in the presence of increasing concentrations of heme ranging from 20 to 170 μM, as well as 500 μM l-ascorbic acid as the electron source to initiate degradation. Reactions containing both PhuS and HemO used 40 μM of each enzyme. The stock heme solution was created by dissolving hemin (Sigma-Aldrich) into 0.5% ethanolamine and diluting it with 25 mM Tris pH 8.5, 100 mM NaCl to a concentration of 3 mM just before addition to the reactions. Eight different heme concentrations were used and their absorbance values measured using a microplate spectrophotometer (PowerWave XS, Bio-Tek, Winooski, VT) at 15-second intervals for a total of 10 minutes after addition of the l-ascorbic acid. Wavelength maxima of 655 nm for verdoheme and 671 nm for biliverdin were used to monitor the amount of product formed by PhuS and HemO, respectively. Reactions containing both PhuS and HemO used 655 or 671 nm, depending on the product monitored. Absorbance values obtained from the microplate spectrophotometer were path length corrected to 1 cm.

### Kinetic Analysis

Following the reaction time, absorbance data were tabulated and graphs of absorbance over time were created for each reaction. Lines of best fit were calculated and the corresponding slope values determined to represent the initial velocity of each reaction in Abs/s at the respective heme concentration. Extinction coefficient values of verdoheme at 655 nm for PhuS and biliverdin at 671 nm for HemO were determined to be 5.17 mM^−1^ cm^−1^ and 4.31 mM^−1^ cm^−1^, respectively, and were used to convert the velocities from Abs/s to mM/s using the Beer-Lambert law. Each enzyme reaction was performed at least five times and the velocity values at each individual heme concentration were averaged to produce a final plot of velocity over heme concentration. Using these curves, the K_m_ and V_max_ values were calculated for both enzymes using GraphPad Prism version 5.0b for Mac OS X, GraphPad Software, La Jolla California USA, www.graphpad.com.

### CO Activity Assay

In 1.5-mL amber vials with screw caps and Chromatherm septa (Chromatographic Specialties Inc., Brockville, ON), 150-μL reaction volumes of 50 μM heme and 0.1 μM PhuS, 0.1 μM HemO, or 0.1 μM PhuS + 0.1 μM HemO in 100 mM potassium phosphate buffer pH 7.0 were equilibrated with constant shaking at 37 °C for 10 minutes, during which time the headspace gas in each vial was purged with CO-free air for 10 seconds. Enzymatic heme degradation was initiated by adding 50 μM l-ascorbic acid, and samples were incubated at 37 °C for 25 minutes with constant shaking. The reactions were stopped by placing the vials on powdered dry ice. The amount of CO in the headspace of each vial was measured using a ta3000R gas chromatograph (Ametek Process Instruments, Newark, DE). The amount of liberated CO was determined by comparing peak area measurements for CO in samples against linear CO standard curves.

### Data Availability

All data generated or analyzed during this study are included in this published article (and its Supplementary Information files).

## Electronic supplementary material

Supplementary Info
