# Supplementary material for: Function Coupling Mechanism of PhuS and HemO in Heme Degradation
Source: Sci Rep. 2017 Sep 12;7:11273. doi: 10.1038/s41598-017-11907-5 (PMC5596023; doi:10.1038/s41598-017-11907-5)

Supporting Information for "Function coupling mechanism of PhuS and HemO in heme degradation"

Michael J.Y. Lee[1,#], Ye Wang[2,#], Yafei Jiang[2], Xichen Li[2], Jianqiu Ma[2], Hongwei Tan[2,*], Keegan Turner-Wood[1], Mona N. Rahman[1], Guangju Chen[2], Zongchao Jia[1,*]

1. Department of Biomedical and Molecular Sciences, Queen's University, Kingston, Ontario, Canada K7L 3N6.
2. College of Chemistry, Beijing Normal University, 100875, Beijing, China.

Supplemental Table S1

Supplemental Figures S1, S2

Structural Figures

- HemO-catalyzed heme hydroxylation
- HemO-catalyzed verdoheme ring cleavage
- PhuS-catalyzed heme hydroxylation

Table S1 Details of Computational models used in this study

Reaction Step	Total Atoms	Atoms in QM region	Spin states of QM region	Charge of QM region
PhuS-heme hydroxylation	5464	150	Singlet Triplet Quintet	+2
HemO-heme hydroxylation	2992	164	Doublet quartet Sextet	+2
HemO-verdoheme cleavage	2991	163	Doublet quartet Sextet	+2

Supplemental Figures

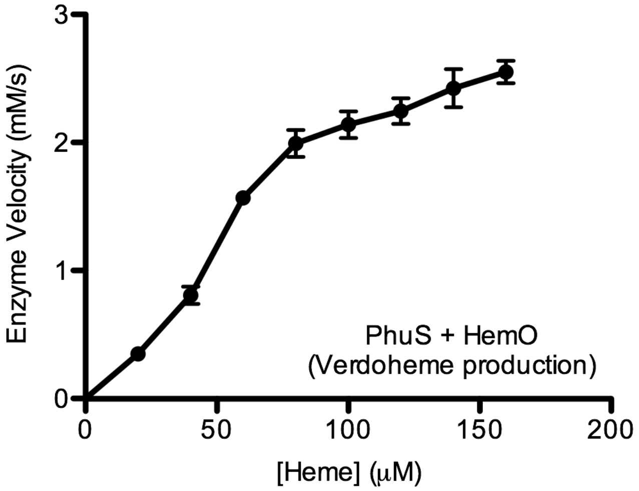

Figure S1. Final velocity plot of verdoheme production for PhuS and HemO together obtained by averaging velocity values of five replicates for each reaction. Each reaction contained 40 μM of both enzymes in the presence of increasing heme concentrations. Error bars represent standard error of the mean. Verdoheme production was monitored at 655 nm.

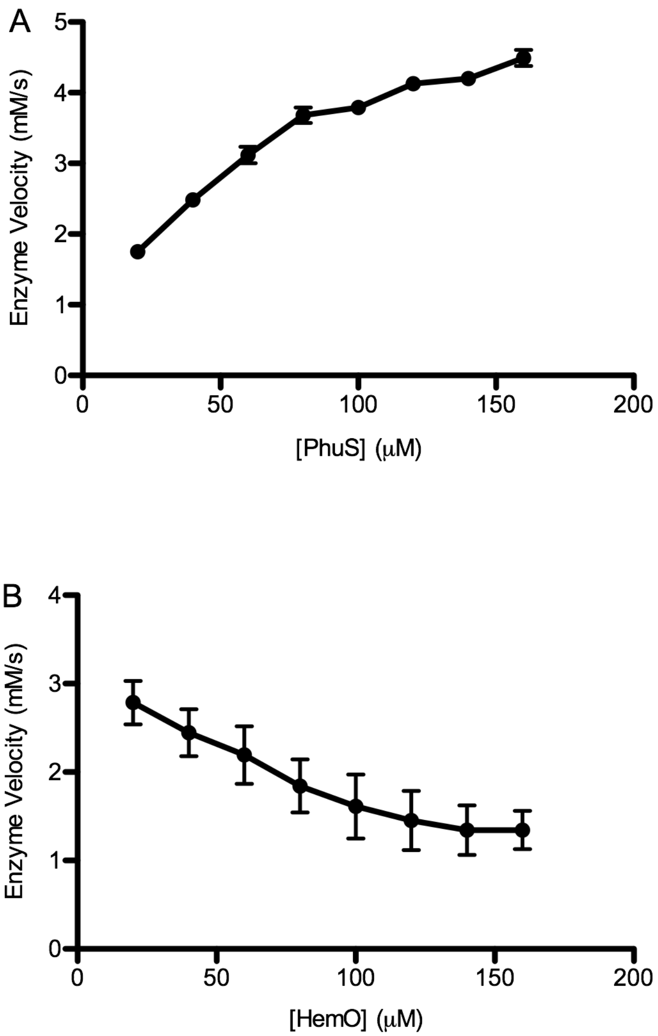

Figure S2. (A) Final velocity plot of biliverdin production in the presence of HemO and increasing concentrations of PhuS obtained by averaging velocity values of replicates for each reaction. Each reaction contained 40 μM HemO, 300 μM heme, and increasing PhuS concentrations. Error bars represent standard error of the mean. (B) Final velocity plot of biliverdin production in the presence of PhuS and increasing concentrations of HemO obtained by averaging velocity values of replicates for each reaction. Each reaction contained 40 μM PhuS, 300 μM heme, and increasing HemO concentrations. Error bars represent standard error of the mean.

HemO-catalyzed heme hydroxylation

Heme[Fe(III)]–O_2H_2 **^{2}A**

E = -4220.37872228 a.u.

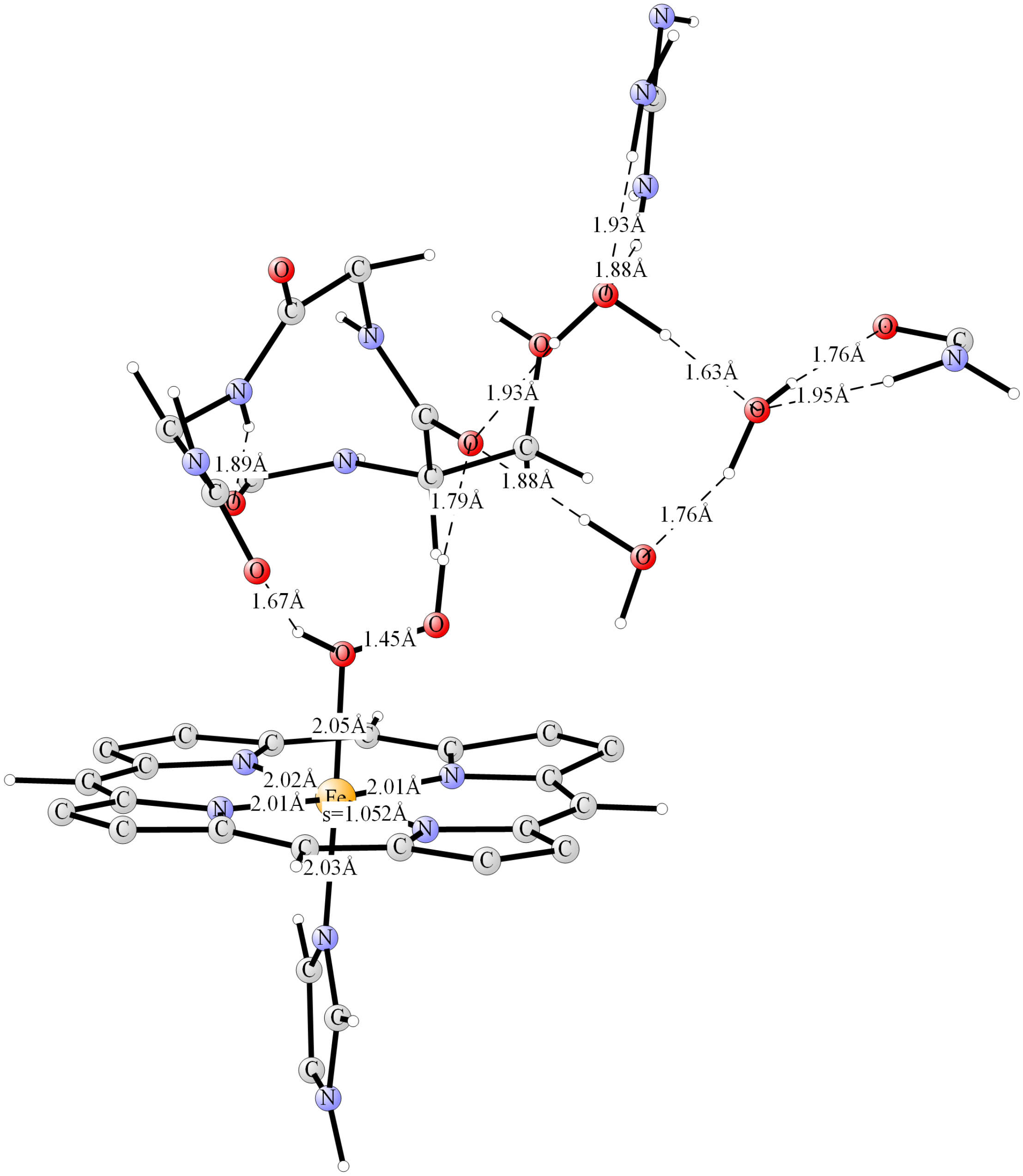

HemO-catalyzed heme hydroxylation

TS $HO_{Fe}\cdots OH$ ^{2}A

E = -4220.34134130 a.u.

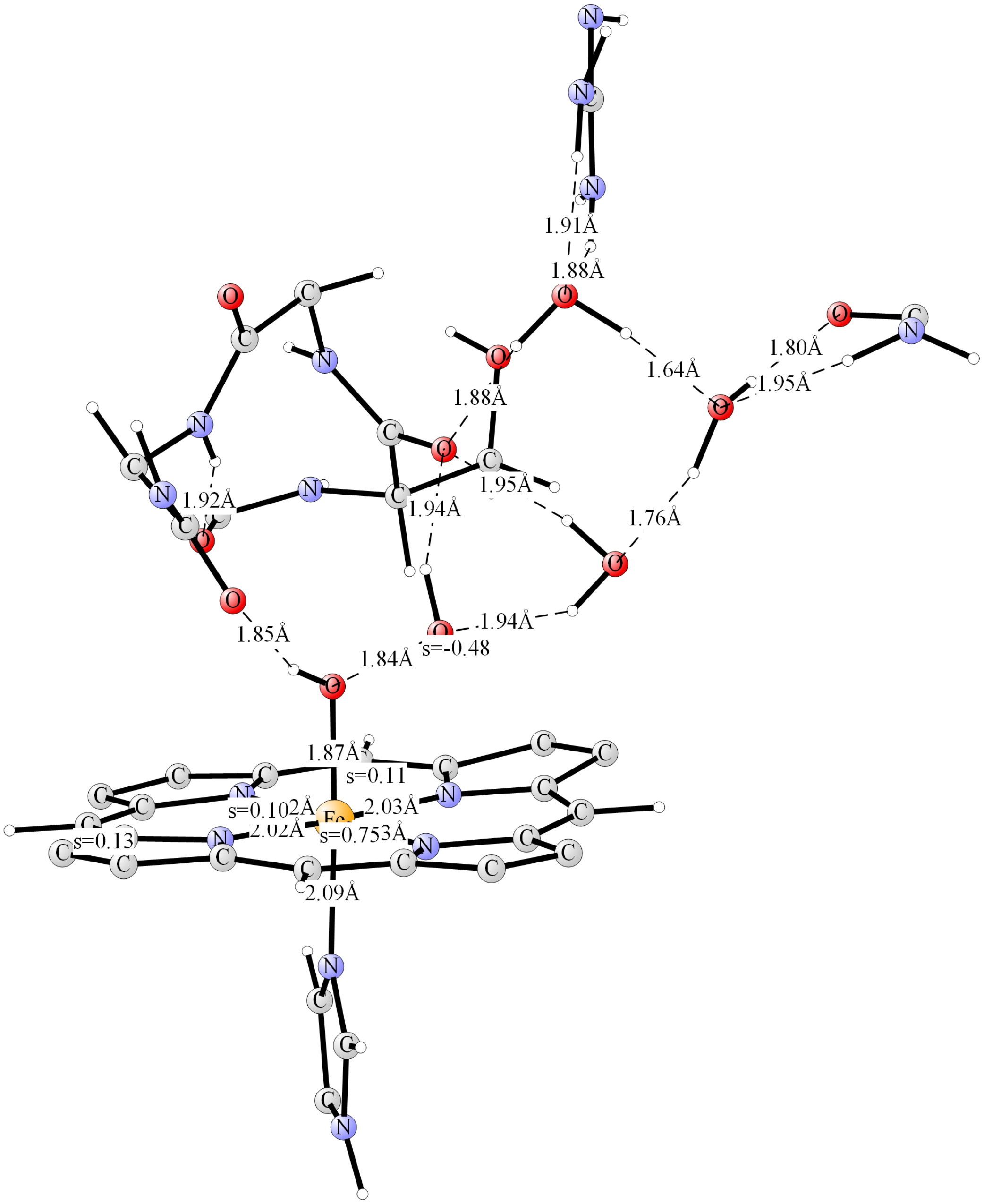

HemO-catalyzed heme hydroxylation

Heme^{+}•[Fe(III)]–OH···OH• **^{2}A**

E = -4220.35084468 a.u.

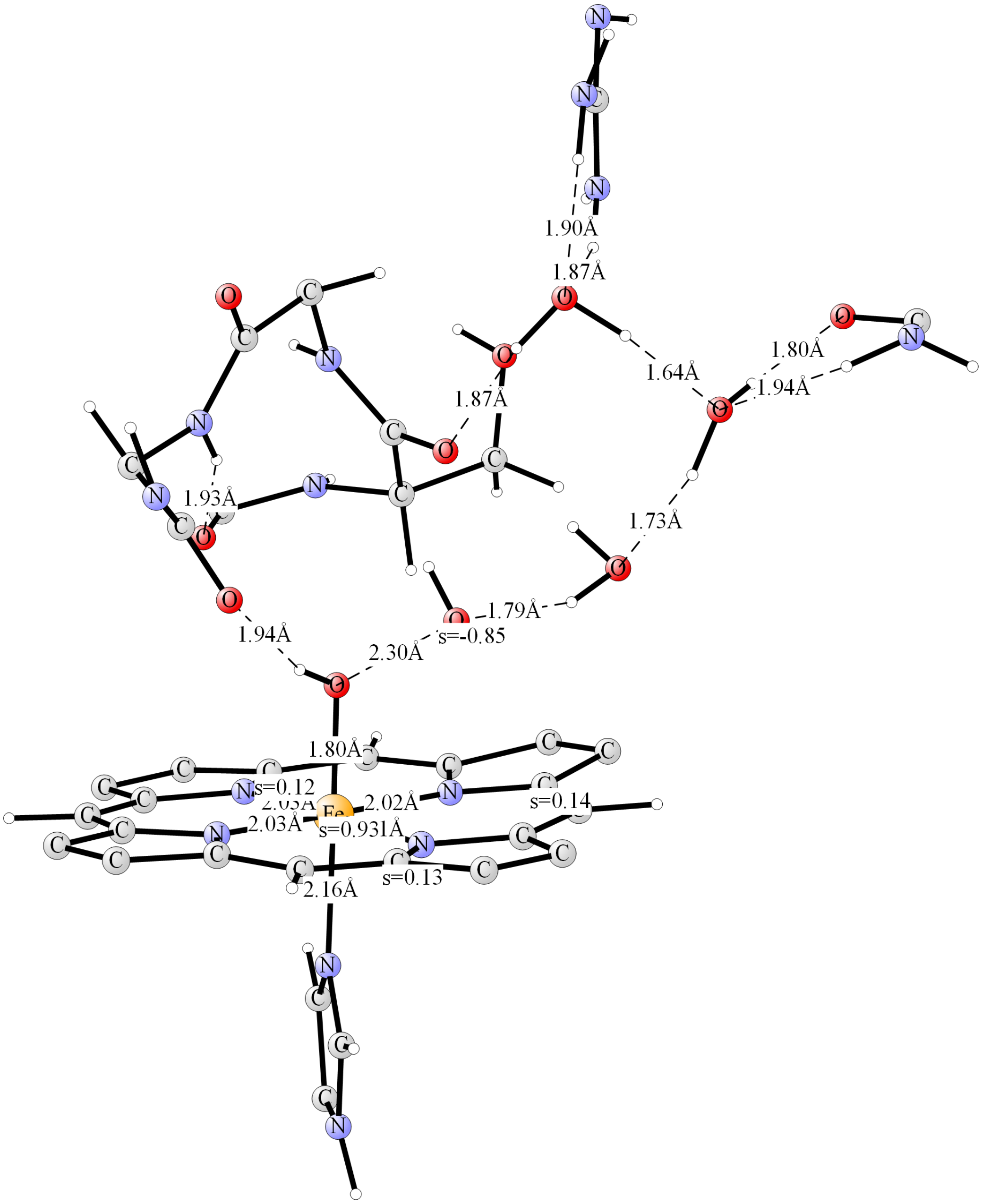

HemO-catalyzed heme hydroxylation

Hydroxyheme[Fe(III)]–OH ^{2}**A**

E = -4220.42681377 a.u.

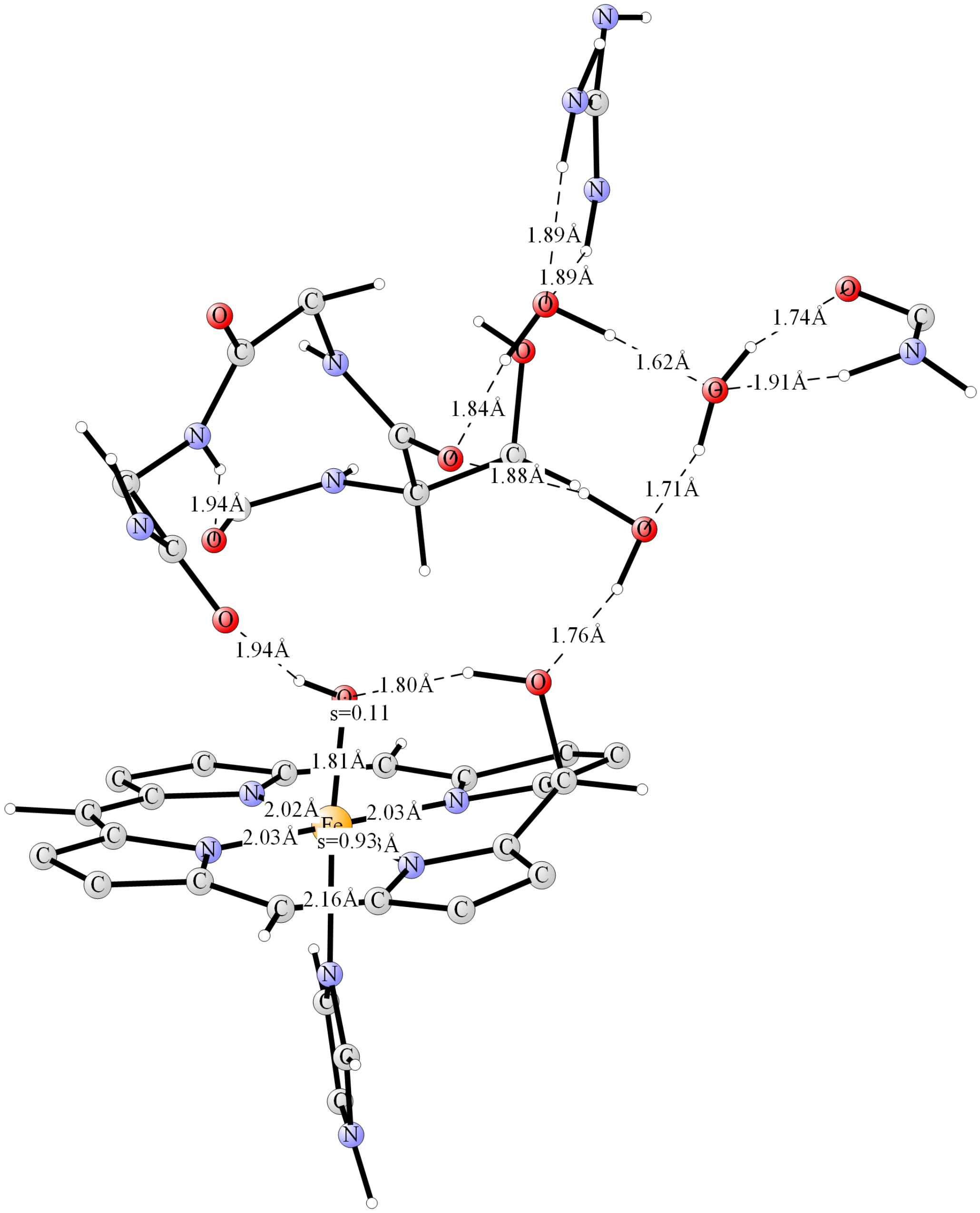

HemO-catalyzed verdoheme ring cleavage

Verdoheme[Fe(II)]–O_2H_2 **^{1}A**

E = -4256.89224756 a.u.

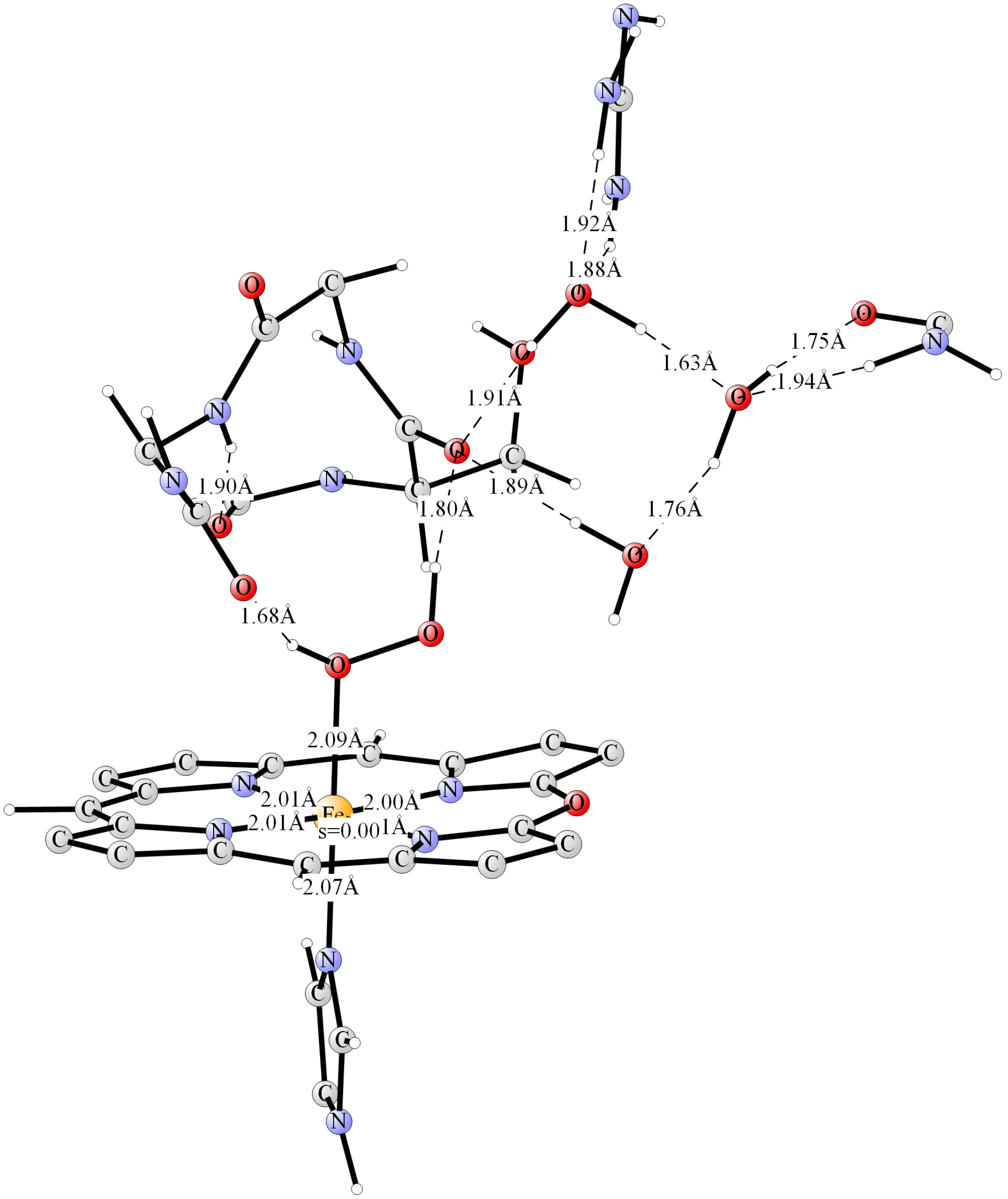

HemO-catalyzed verdoheme ring cleavage

TS HO$_{Fe}$···OH ^{1}**A**

E = -4256.87077588 a.u.

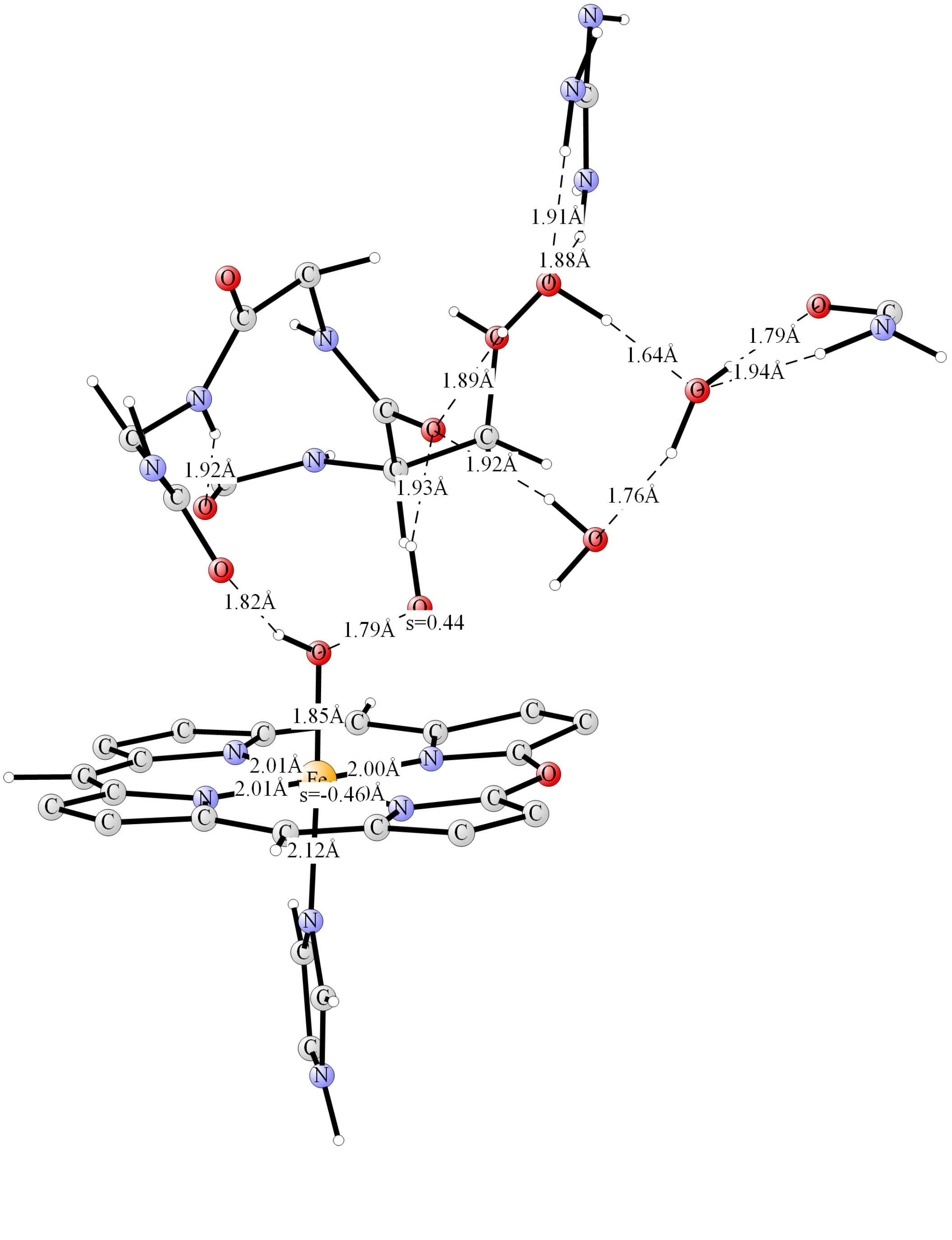

HemO-catalyzed verdoheme ring cleavage

Verdoheme[Fe(III)]–OH···OH• ^{3}**A**

E = -4256.88529916 a.u.

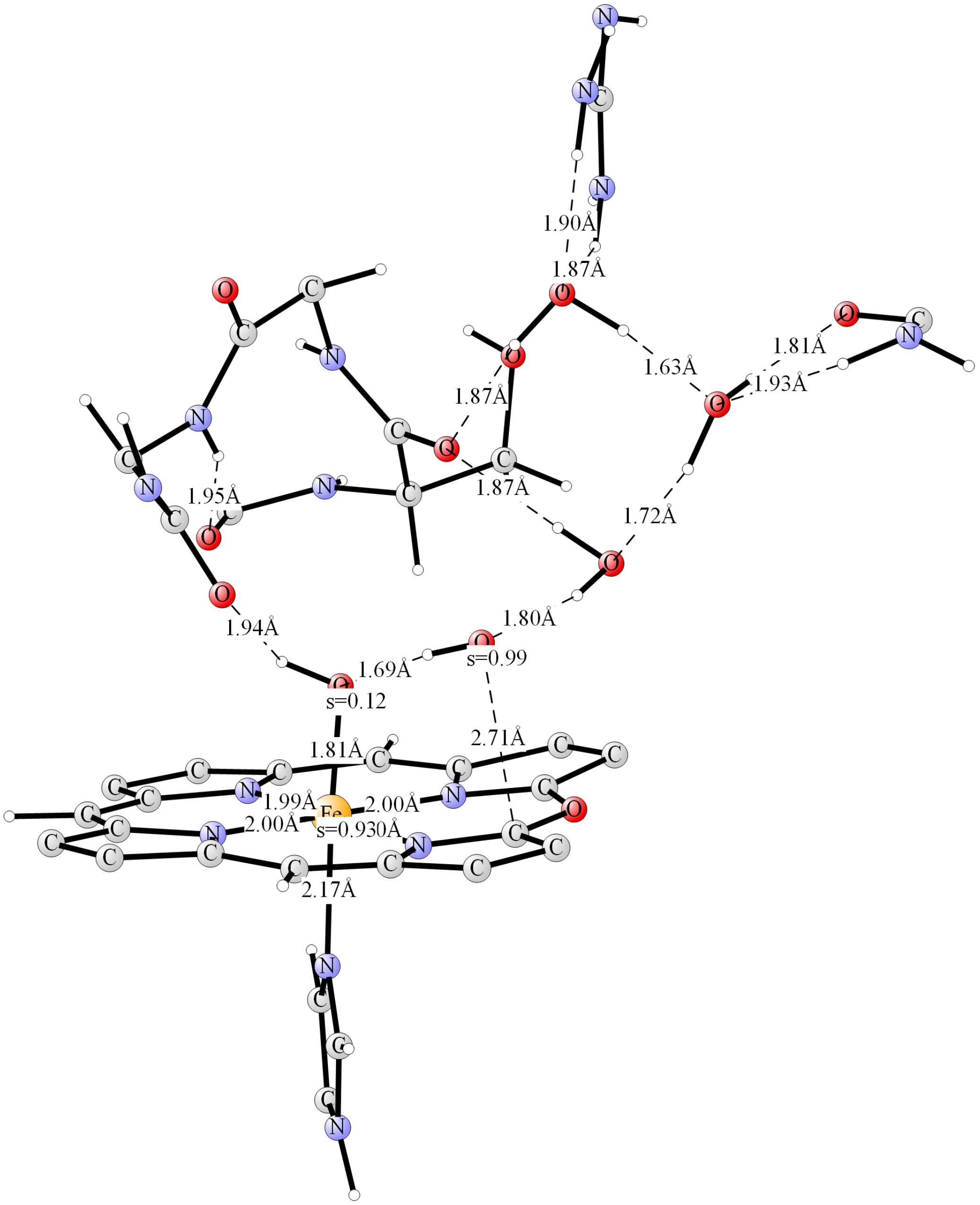

HemO-catalyzed verdoheme ring cleavage

TS $C_{Verdoheme}\cdots OH\bullet$ **^{3}A**

E = -4256.88357911 a.u.

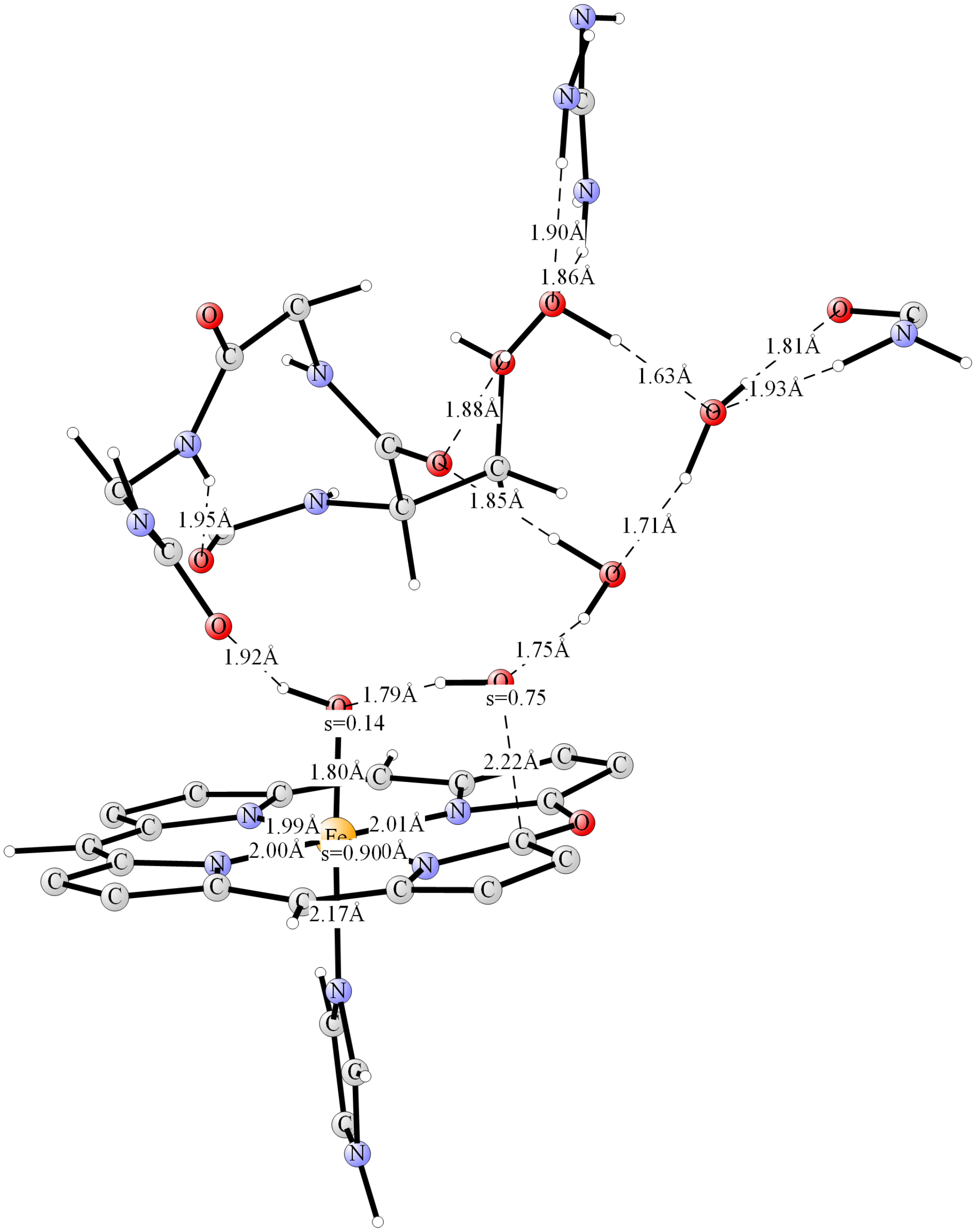

HemO-catalyzed verdoheme ring cleavage

Hydroxyverdoheme[Fe(III)]–OH **^{3}A**

E = -4256.94183941 a.u.

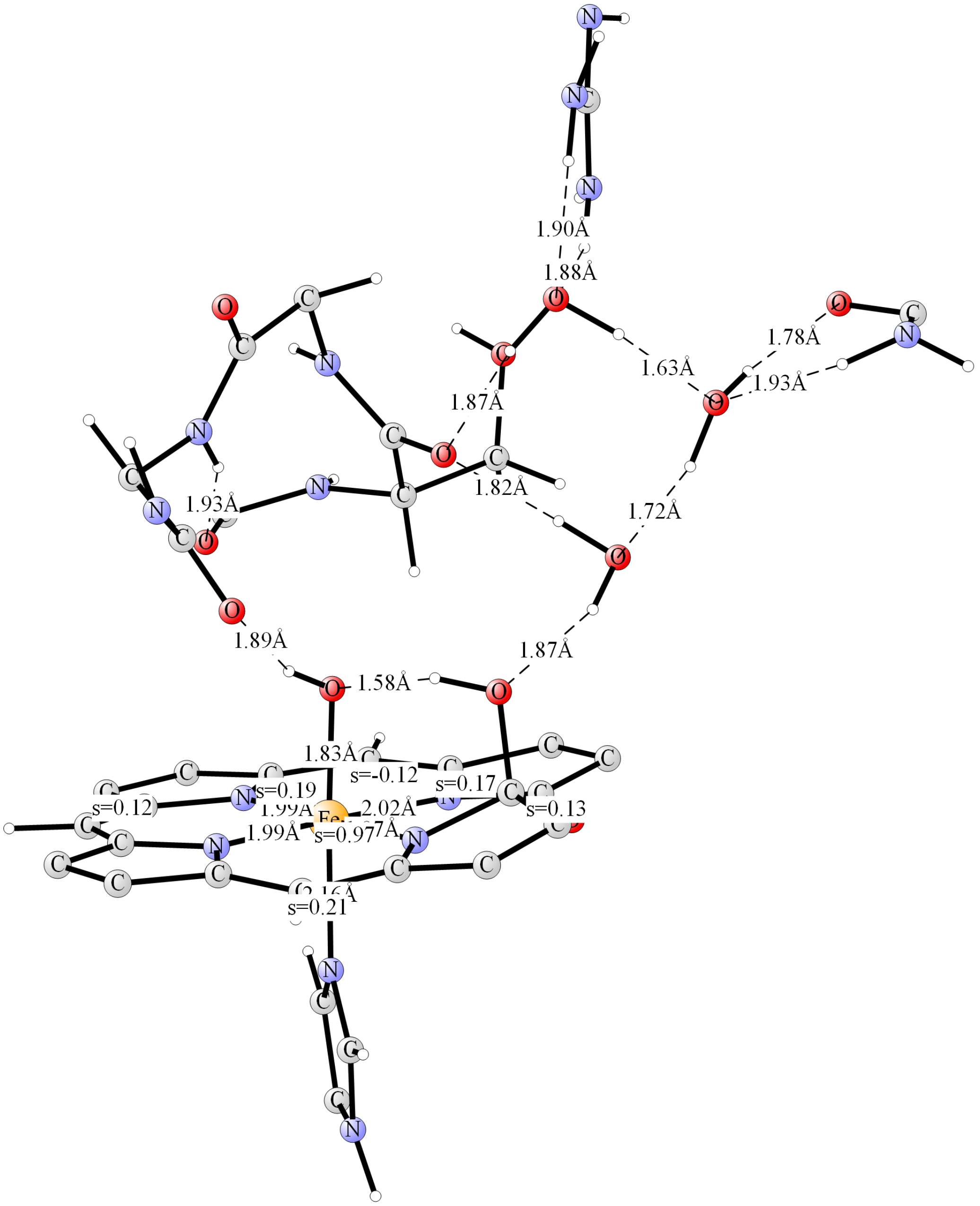

HemO-catalyzed verdoheme ring cleavage

TS $HO_{Fe}\cdots H\cdots O_{Hydroxyverdoheme}$ **^3A**

E = -4256.93921328 a.u.

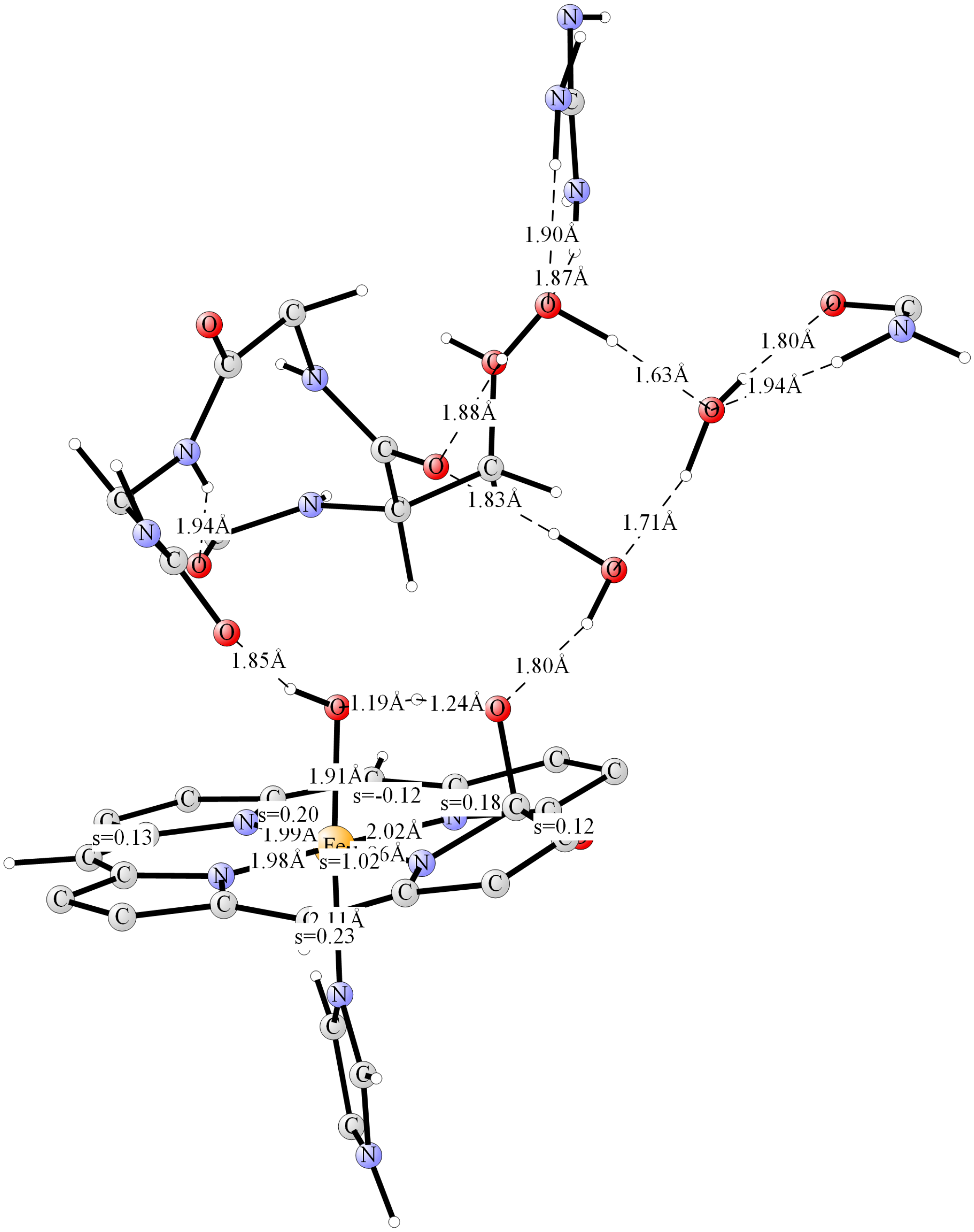

HemO-catalyzed verdoheme ring cleavage

Hydroxyverdoheme^{-}•[Fe(III)]–OH$_2$ ^{3}**A**

E = -4256.94099694 a.u.

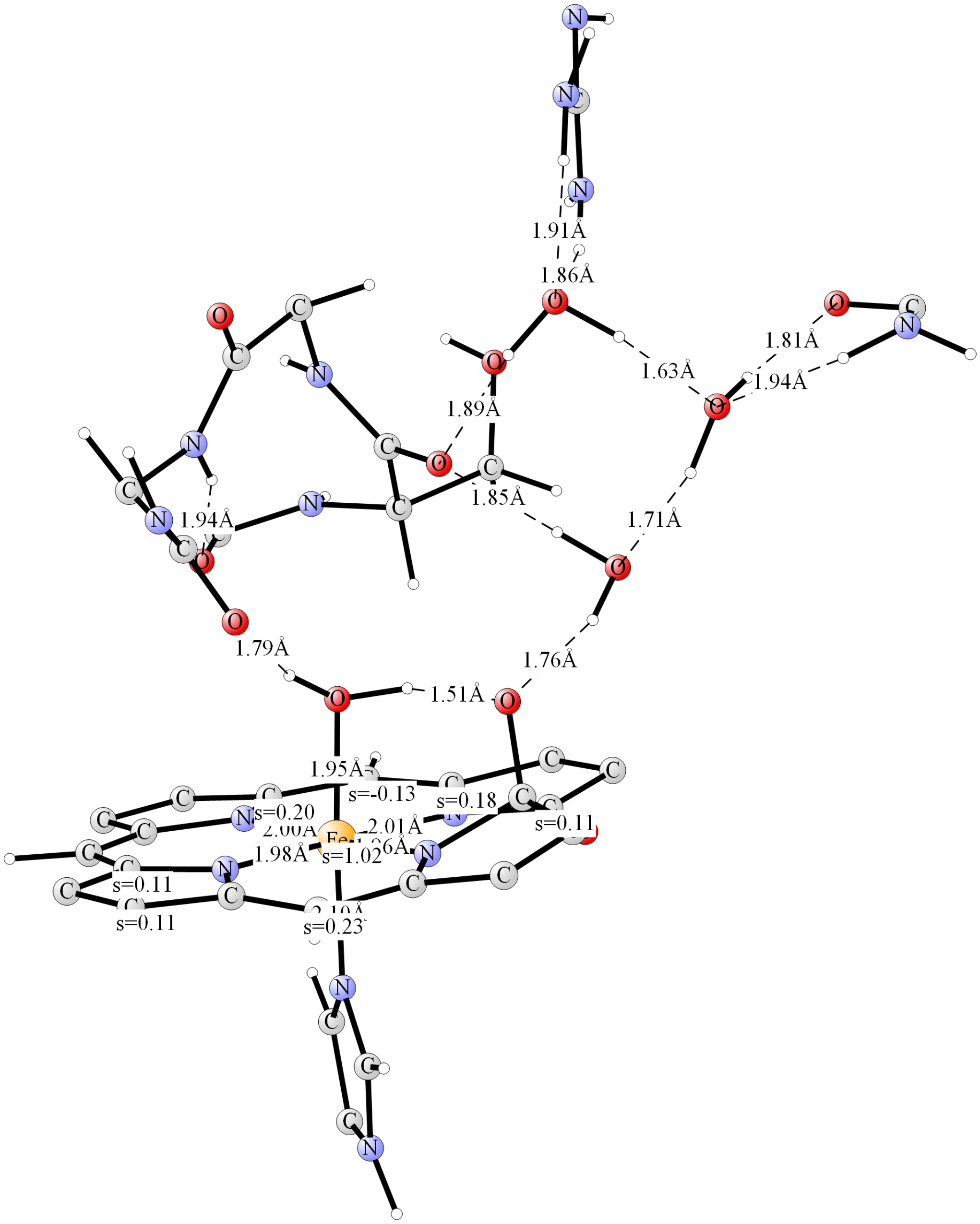

HemO-catalyzed verdoheme ring cleavage

TS $C_{Hydroxyverdoheme\text{-}\bullet}\cdots O_{Hydroxyverdoheme\text{-}\bullet}$ **^{3}A**

E = -4256.93817376 a.u.

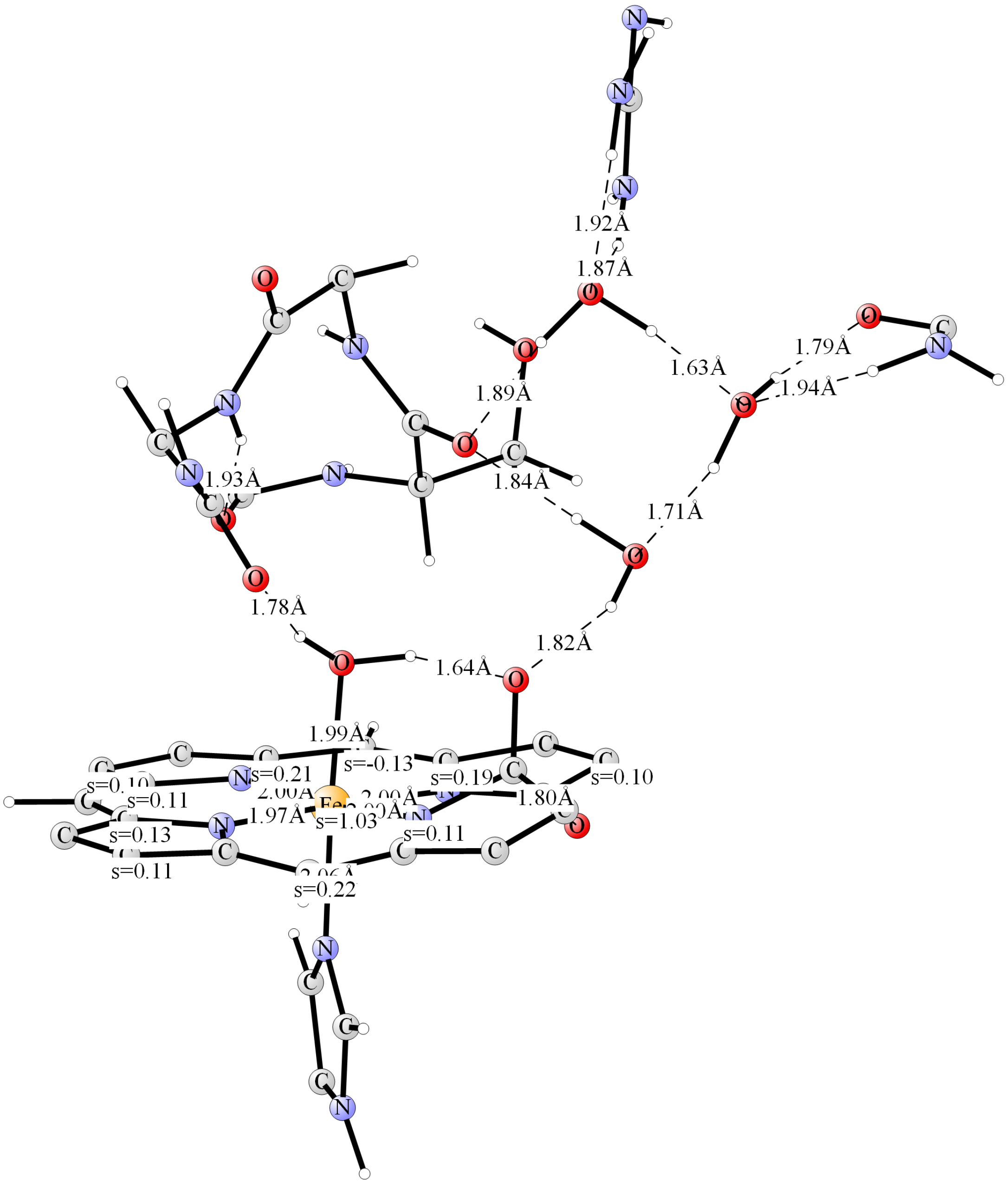

HemO-catalyzed verdoheme ring cleavage

Hydroxyverdoheme^{-}•[Fe(III)]–OH$_2$ cleaved **^{3}A**

E = -4256.95161630 a.u.

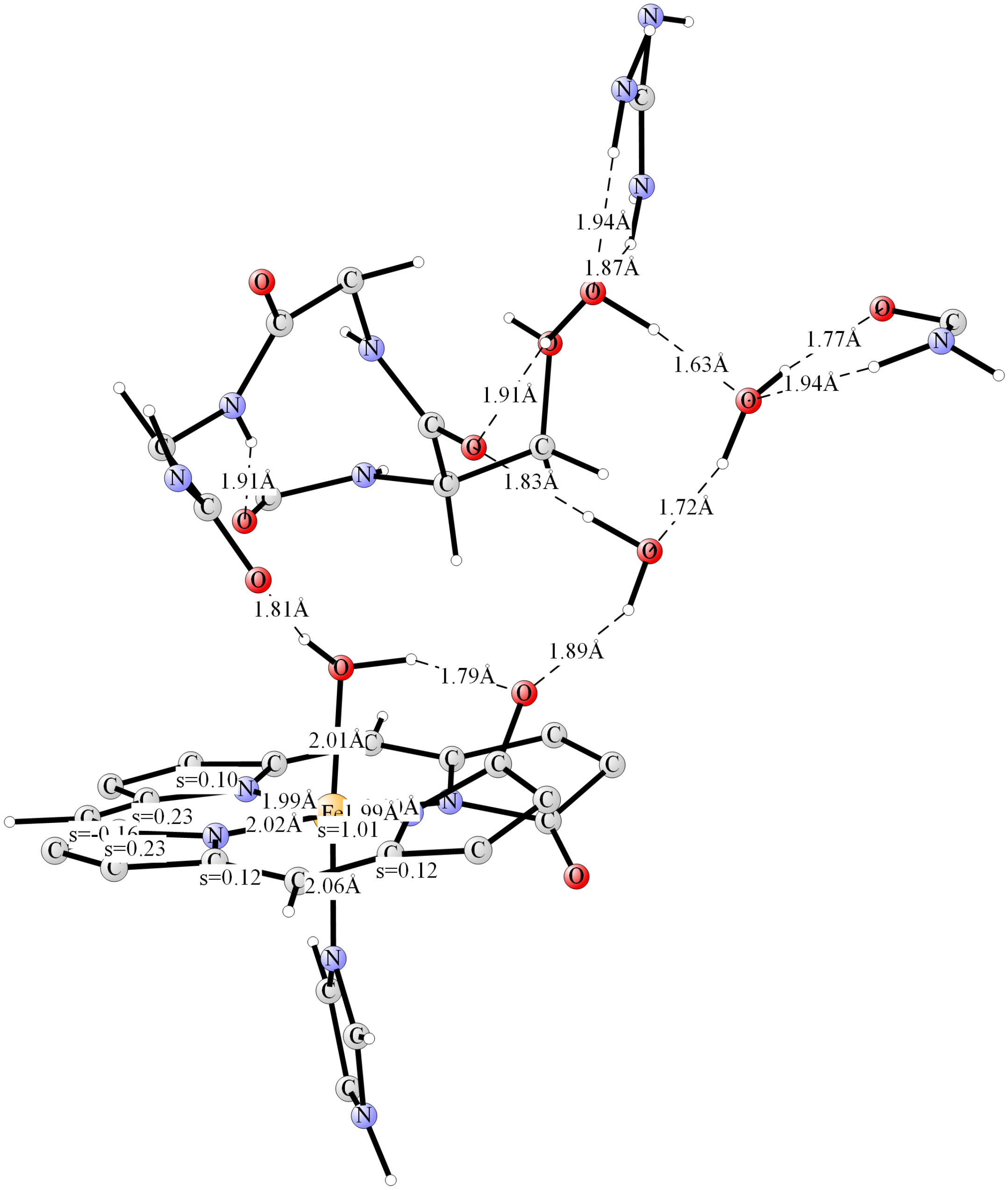

PhuS-catalyzed heme hydroxylation

Heme[Fe(III)]–O_2H_2 **^{2}A**

E = -3761.99827963 a.u.

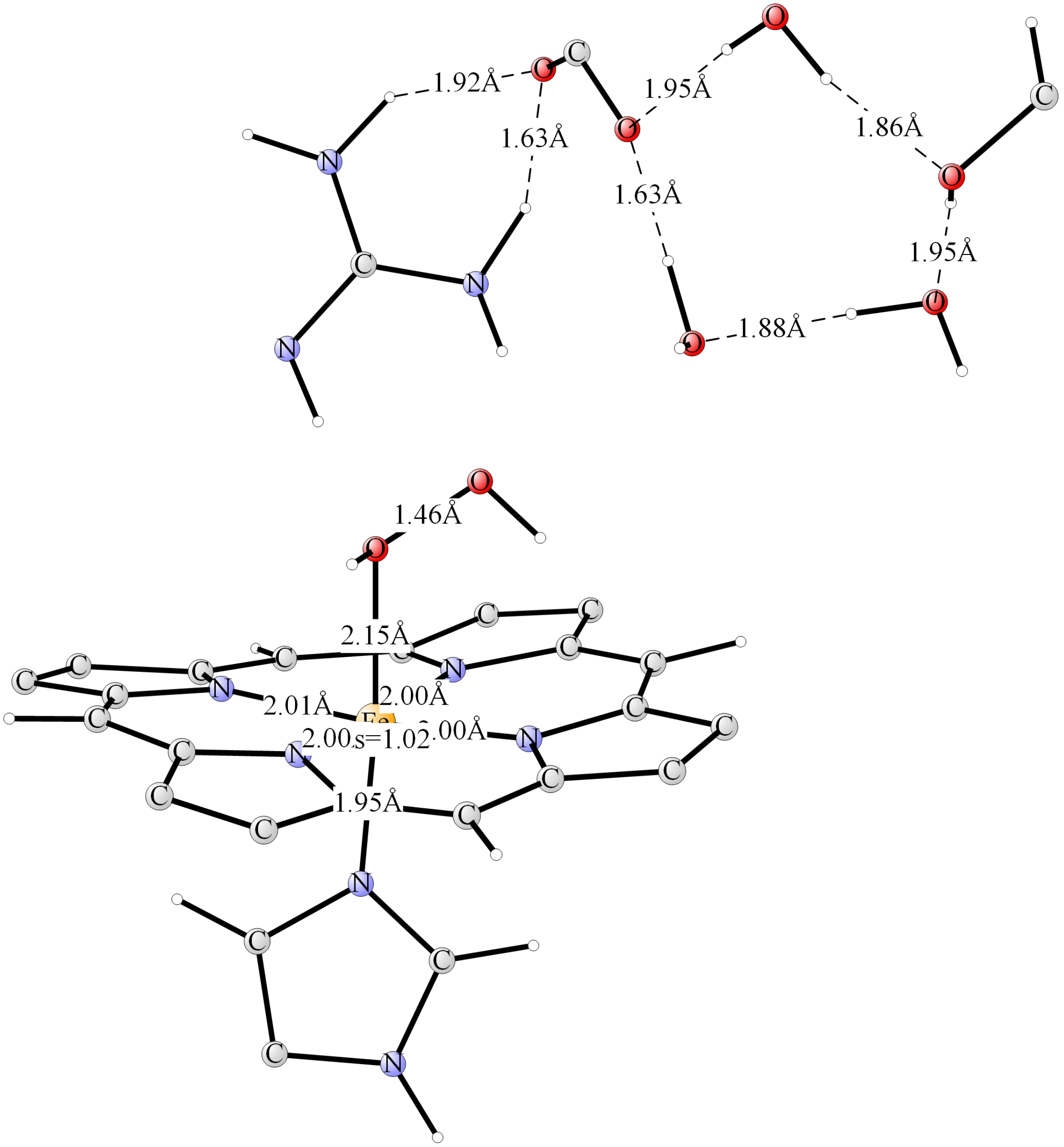

PhuS-catalyzed heme hydroxylation

TS $HO_{Fe}\cdots OH$ **^{2}A**

E = -3761.97341357 a.u.

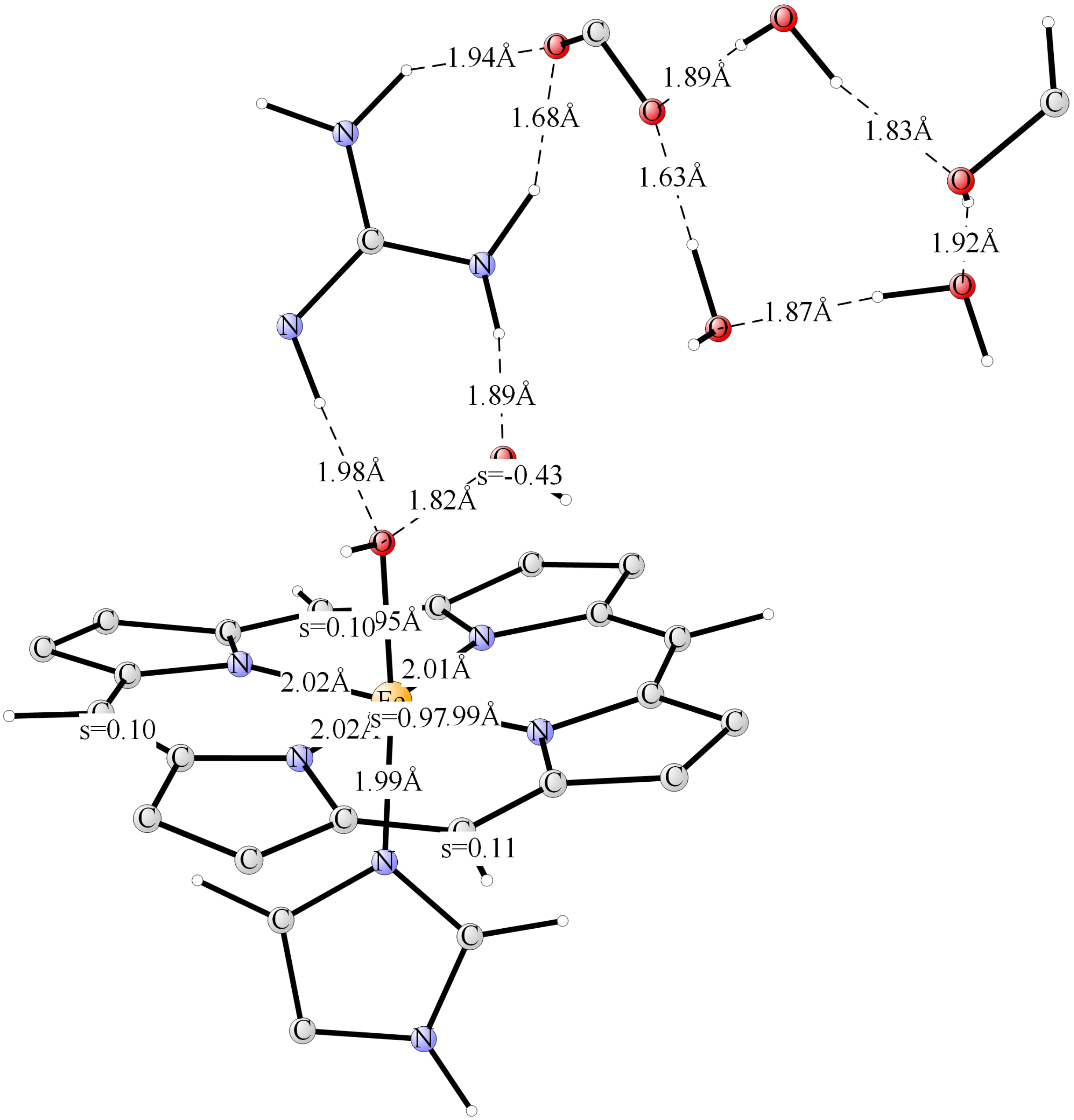

PhuS-catalyzed heme hydroxylation

Heme^{+}•[Fe(III)]–OH···OH• ^{2}**A**

E = -3761.99870290 a.u.

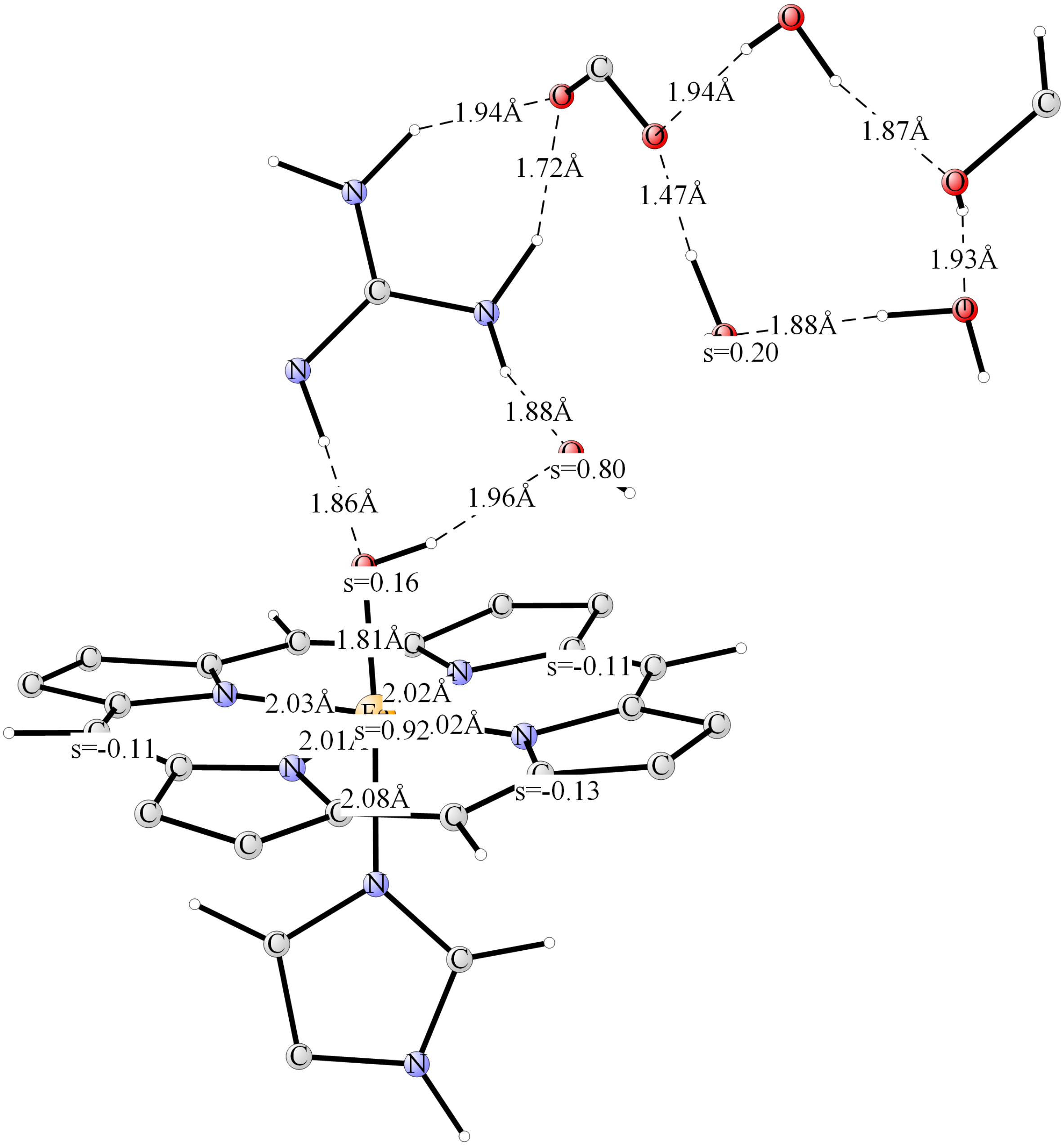

PhuS-catalyzed heme hydroxylation

Hydroxyheme[Fe(III)]–OH **^{2}A**

E = -3762.06774967 a.u.

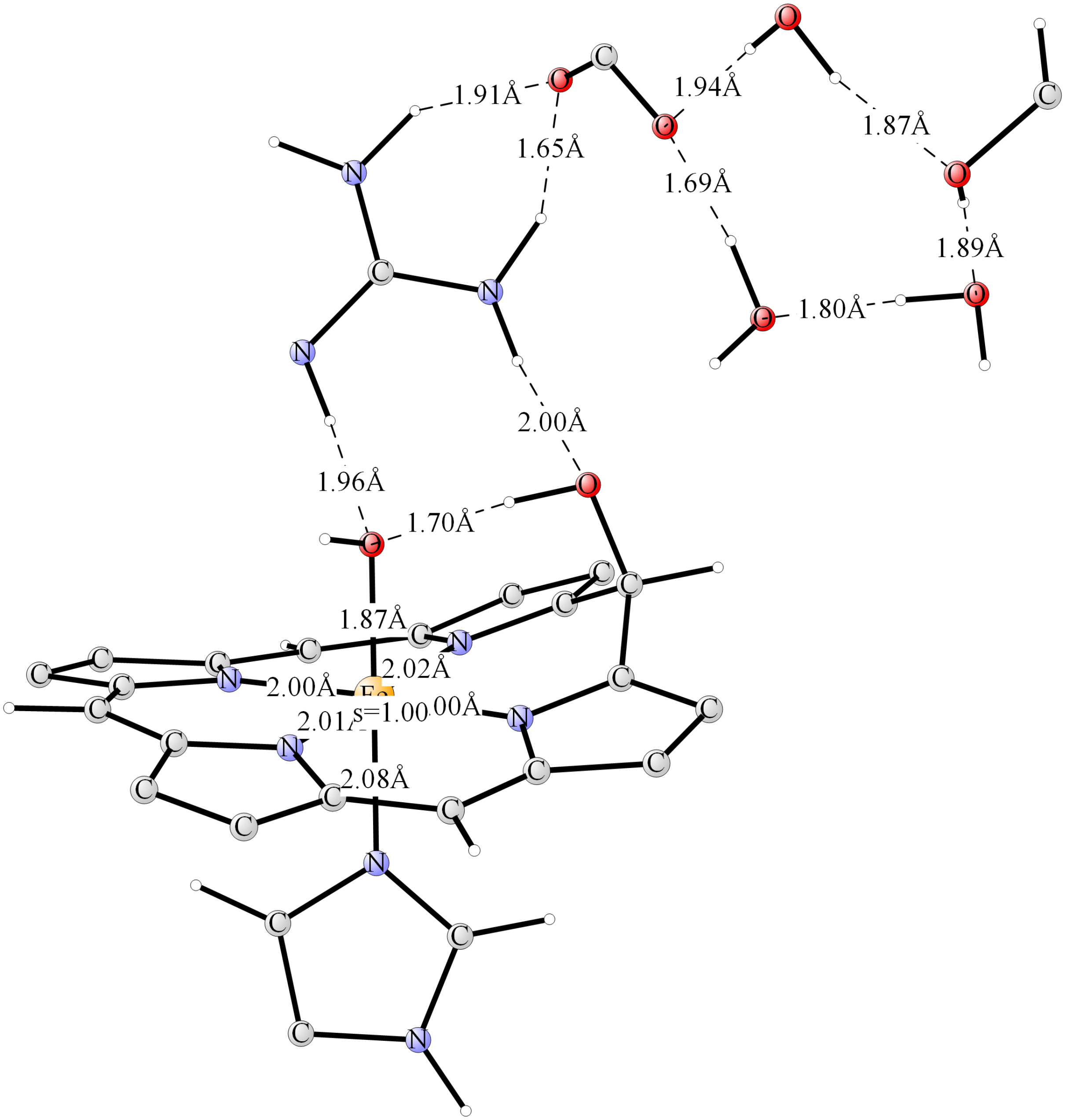

PhuS-catalyzed heme hydroxylation

Heme[Fe(III)]–O_2H_2 **^{4}A**

E = -3761.98468447 a.u.

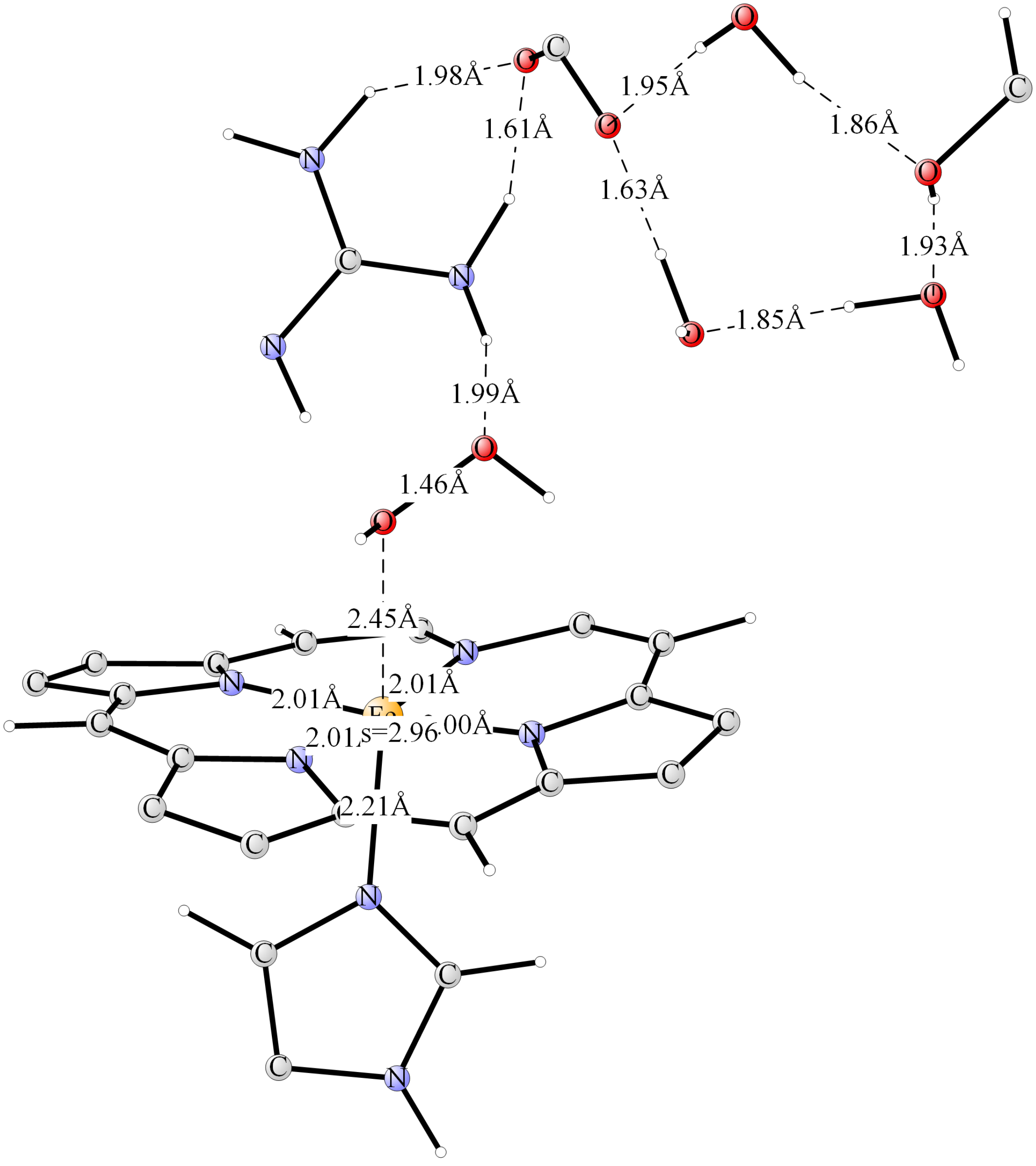

PhuS-catalyzed heme hydroxylation

TS HO$_{Fe}$···OH **^{4}A**

E = -3761.96728239 a.u.

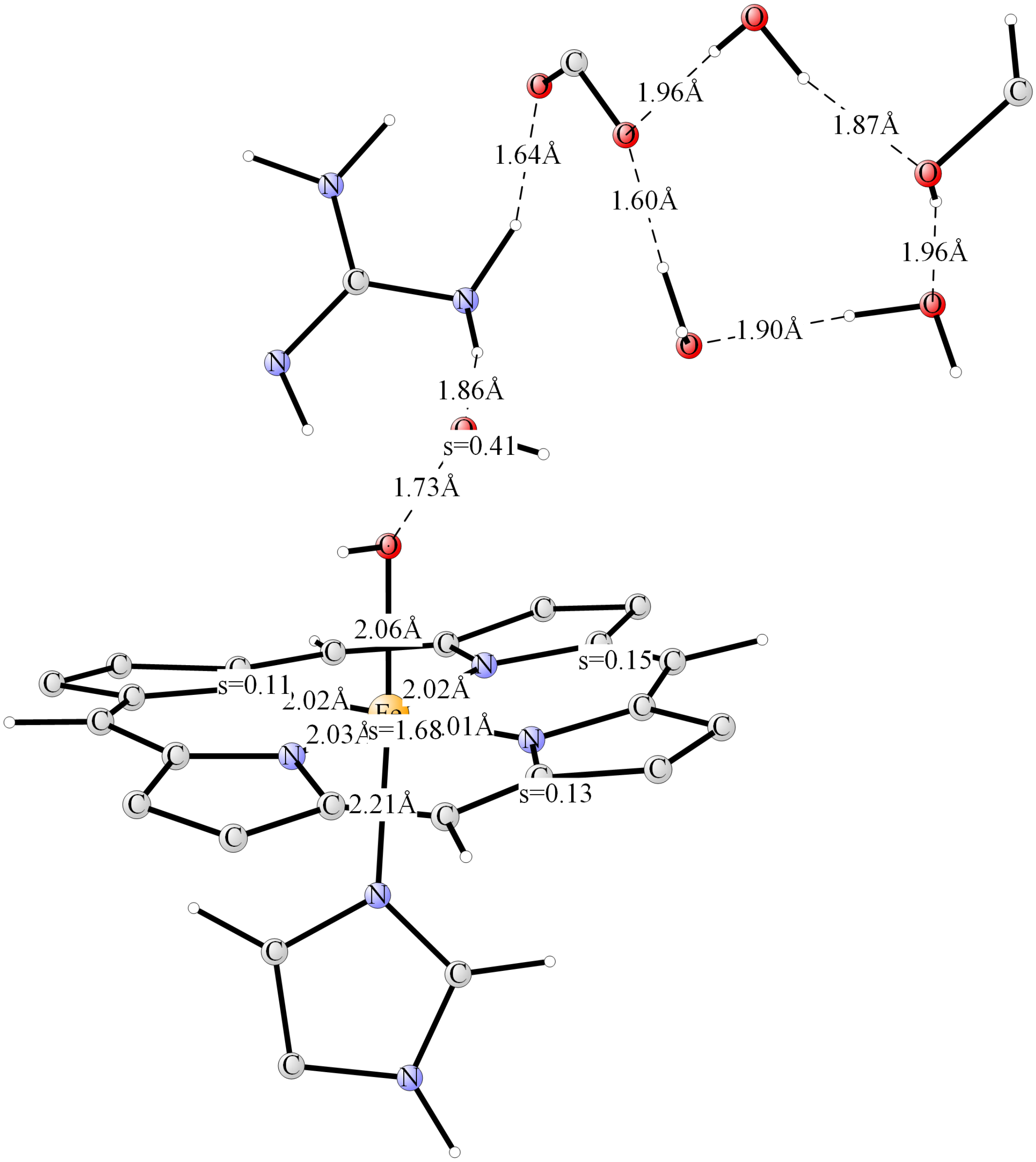

PhuS-catalyzed heme hydroxylation

Heme+•[Fe(III)]–OH···OH• **^{4}A**

E = -3762.00147662 a.u.

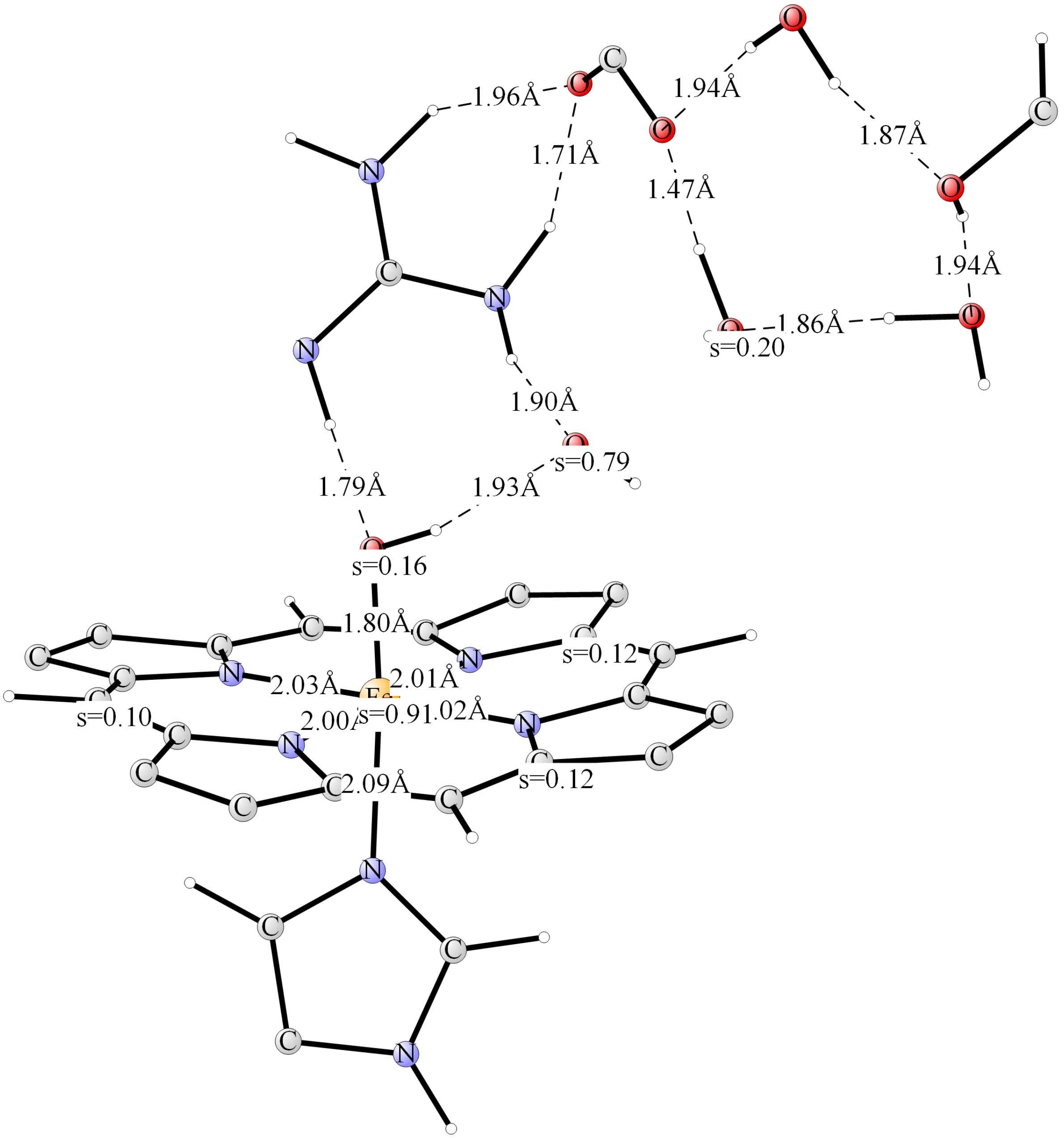

PhuS-catalyzed heme hydroxylation

Hydroxyheme[Fe(III)]–OH **^{4}A**

E = -3762.05468316 a.u.

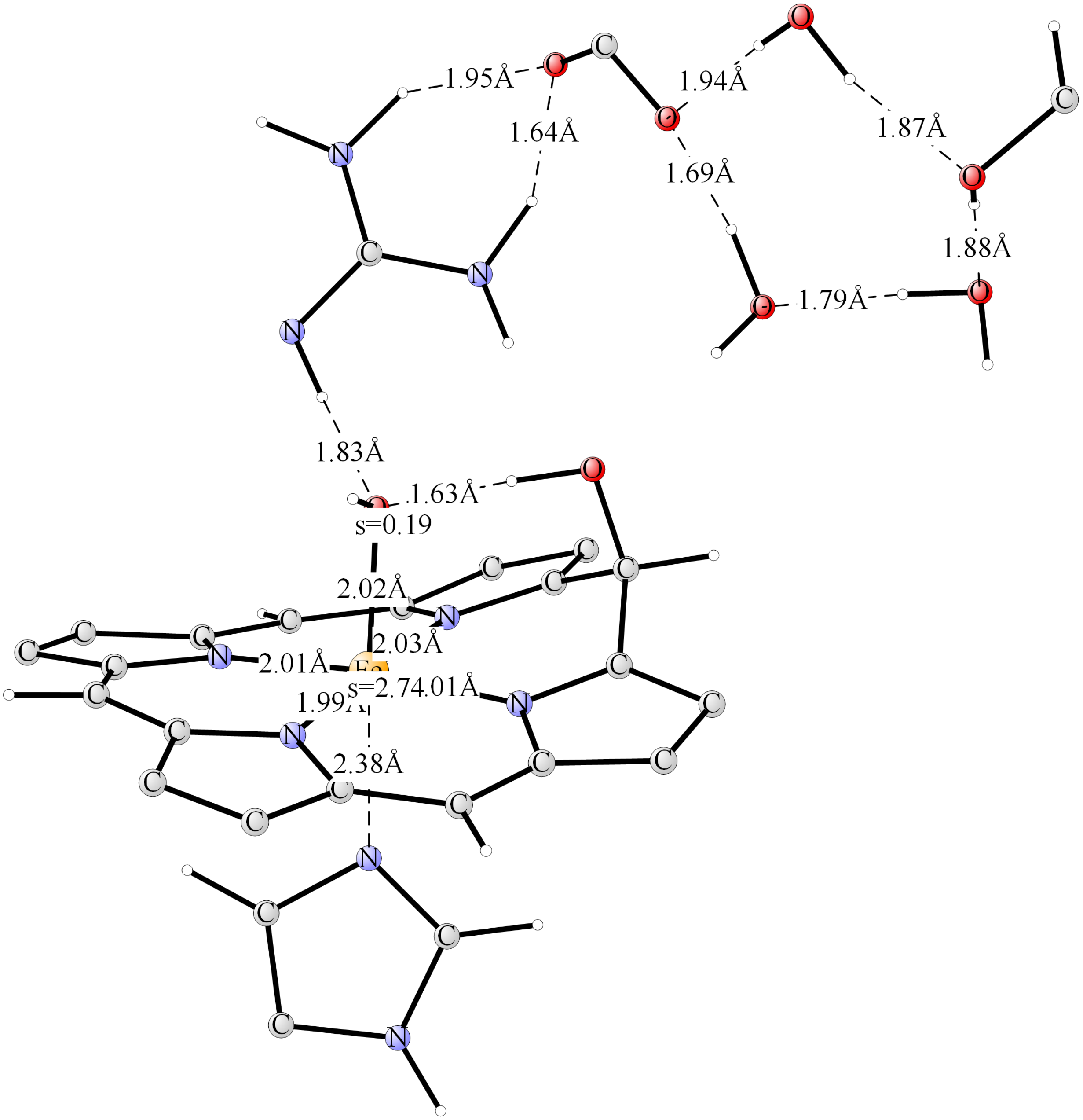

PhuS-catalyzed heme hydroxylation

Heme[Fe(III)]–O_2H_2 **^6A**

E = -3761.98995115 a.u.

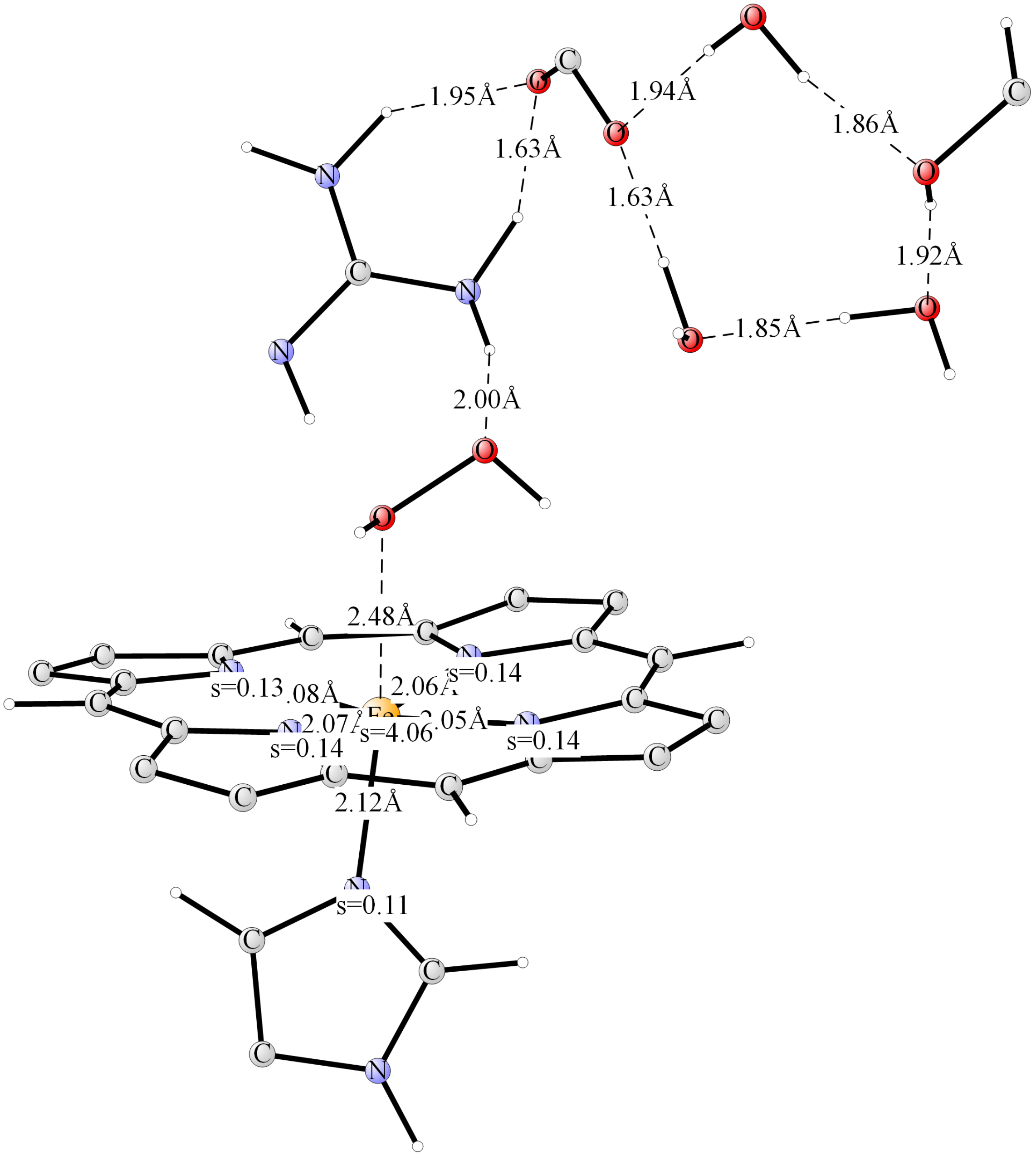

PhuS-catalyzed heme hydroxylation

TS $HO_{Fe}\cdots OH$ ^{6}**A**

E = -3761.95398677 a.u.

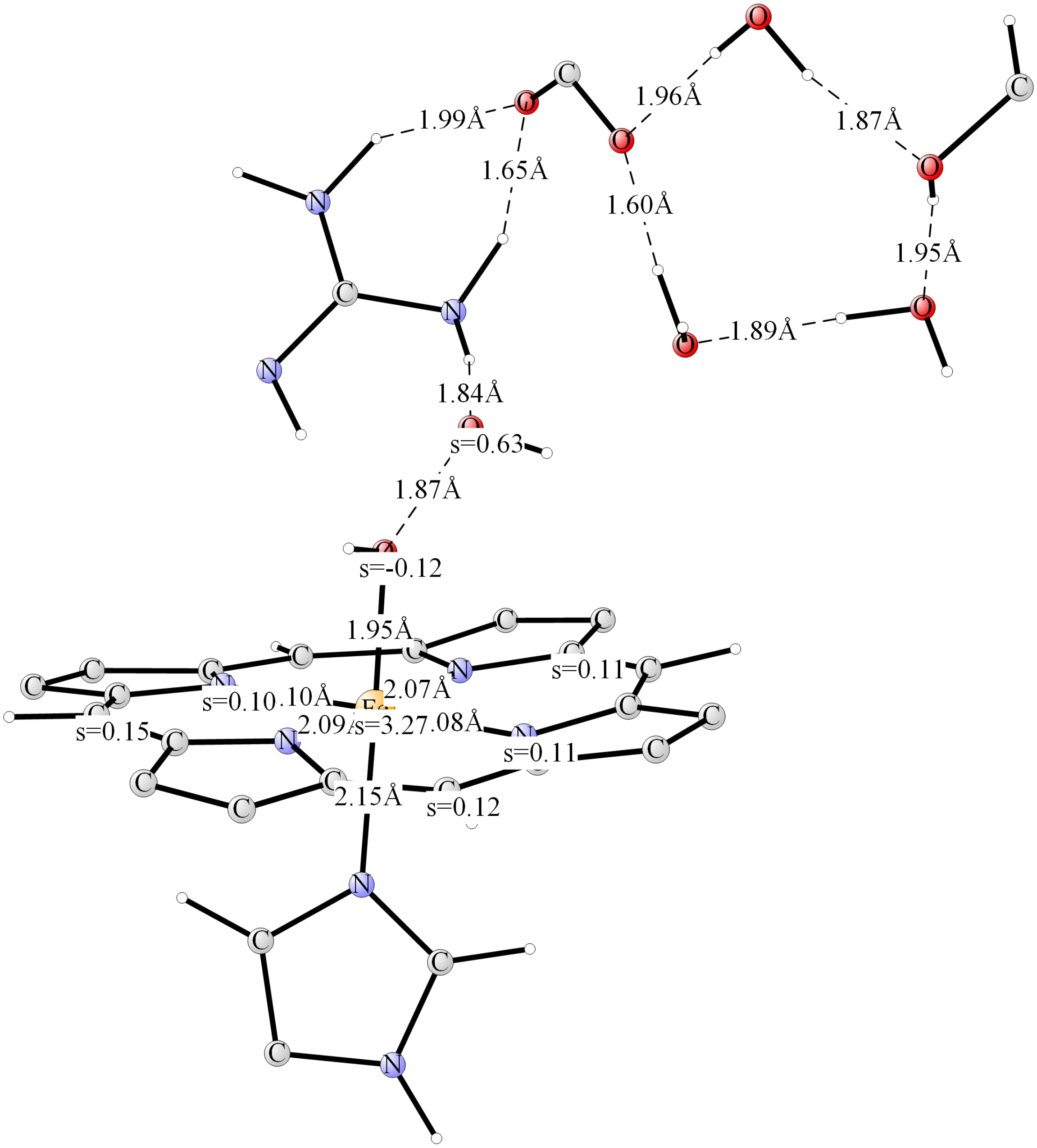

PhuS-catalyzed heme hydroxylation

Heme^{+}•[Fe(III)]–OH···OH• **^{6}A**

E = -3761.97541052 a.u.

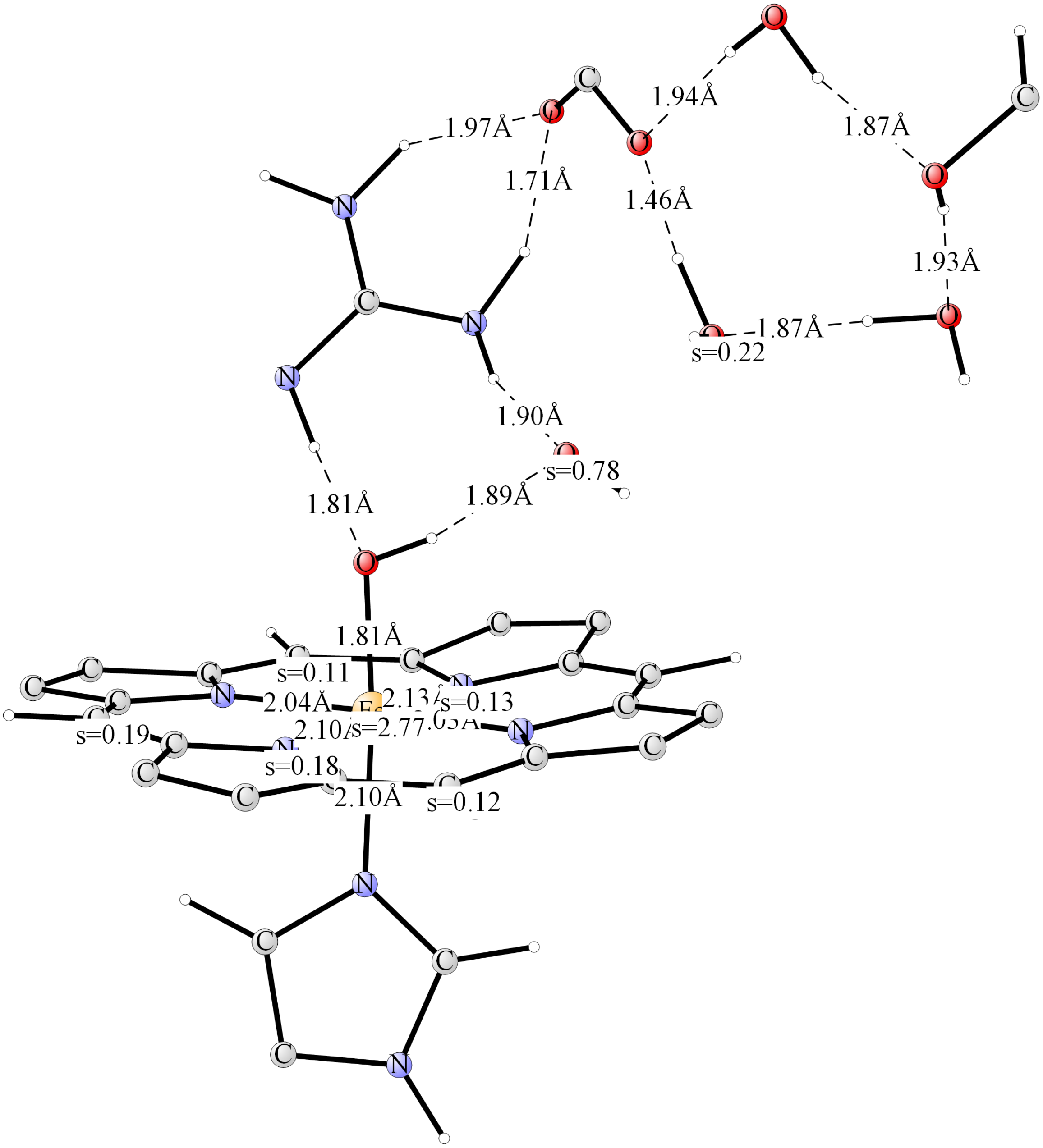

PhuS-catalyzed heme hydroxylation

Hydroxyheme[Fe(III)]–OH **^{6}A**

E = -3762.03522001 a.u.

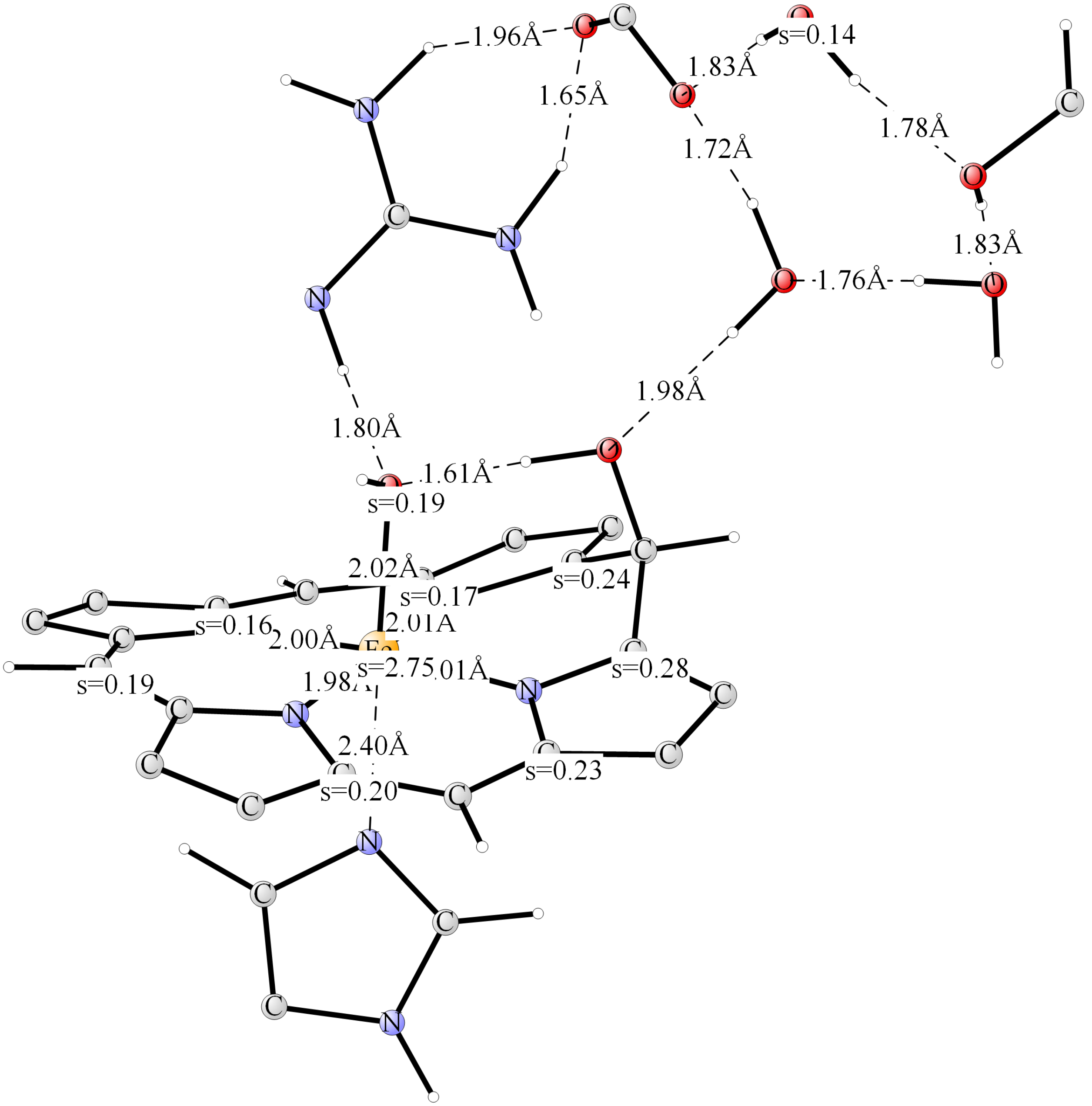

Supplement: Supplementary file 1 — Supplementary Info [file 41598_2017_11907_MOESM1_ESM.pdf]
